# Oncogenic Kinase Cascades Induce Molecular Mechanisms That Protect Leukemic Cell Models from Lethal Effects of De Novo dNTP Synthesis Inhibition

**DOI:** 10.3390/cancers13143464

**Published:** 2021-07-10

**Authors:** Miriam Pons, Yanira Zeyn, Stella Zahn, Nisintha Mahendrarajah, Brent D. G. Page, Patrick T. Gunning, Richard Moriggl, Walburgis Brenner, Falk Butter, Oliver H. Krämer

**Affiliations:** 1 Department of Toxicology, University Medical Center, 55131 Mainz, Germany; yanira.zeyn@uni-mainz.de (Y.Z.); s2254711@stud.uni-frankfurt.de (S.Z.); nisintha@yahoo.de (N.M.); 2 Faculty of Pharmaceutical Science, University of British Columbia, Vancouver, BC V6T 1Z4, Canada; brent.page@ubc.ca; 3 Department of Chemical & Physical Sciences, University of Toronto, Mississauga, ON L5L 1C6, Canada; patrick.gunning@utoronto.ca; 4 Department of Chemistry, University of Toronto, Toronto, ON M5S 3H6, Canada; 5 Institute of Animal Breeding and Genetics, University of Veterinary Medicine, 1210 Vienna, Austria; richard.moriggl@vetmeduni.ac.at; 6 Clinic for Obstetrics and Women’s Health, University Medical Center, 55131 Mainz, Germany; brenner@uni-mainz.de; 7 Institute of Molecular Biology (IMB), 55128 Mainz, Germany; F.Butter@imb-mainz.de

**Keywords:** AML, apoptosis, BCR-ABL1, CML, hydroxyurea, replication stress, RAF1, FLT3, BCL-XL

## Abstract

**Simple Summary:**

Leukemic cells show differential sensitivity to apoptosis induction by the clinically relevant drug hydroxyurea. Since resistance to hydroxyurea can pose a therapeutic problem, we searched for mechanisms that protect such cells from the toxic effects of hydroxyurea. We used proteomics followed by mass spectrometry to accomplish this task and noted a loss of the RAF1 kinase in cells that are killed by hydroxyurea. Pharmacological inhibition of RAF1 and its target BCL-XL show that these proteins suppress apoptosis induction. Furthermore, inhibition of their upstream regulators BCR-ABL1 (in chronic myeloid leukemia cells) and FLT3-ITD (in acute myeloid leukemia cells) plus hydroxyurea produced favorable results. This approach may benefit patients that are not successfully treated with tyrosine kinase inhibitors. Taken together, we provide novel insights into strategies that eliminate chronic and acute myeloid leukemia cells with combinations of clinically established and currently tested pharmaceutical agents.

**Abstract:**

The ribonucleotide reductase inhibitor hydroxyurea suppresses de novo dNTP synthesis and attenuates the hyperproliferation of leukemic blasts. Mechanisms that determine whether cells undergo apoptosis in response to hydroxyurea are ill-defined. We used unbiased proteomics to uncover which pathways control the transition of the hydroxyurea-induced replication stress into an apoptotic program in chronic and acute myeloid leukemia cells. We noted a decrease in the serine/threonine kinase RAF1/c-RAF in cells that undergo apoptosis in response to clinically relevant doses of hydroxyurea. Using the RAF inhibitor LY3009120, we show that RAF activity determines the sensitivity of leukemic cells toward hydroxyurea. We further disclose that pharmacological inhibition of the RAF downstream target BCL-XL with the drug navitoclax and RNAi combine favorably with hydroxyurea against leukemic cells. BCR-ABL1 and hyperactive FLT3 are tyrosine kinases that causally contribute to the development of leukemia and induce RAF1 and BCL-XL. Accordingly, the ABL inhibitor imatinib and the FLT3 inhibitor quizartinib sensitize leukemic cells to pro-apoptotic effects of hydroxyurea. Moreover, hydroxyurea and navitoclax kill leukemic cells with mutant FLT3 that are resistant to quizartinib. These data reveal cellular susceptibility factors toward hydroxyurea and how they can be exploited to eliminate difficult-to-treat leukemic cells with clinically relevant drug combinations.

## 1. Introduction

Chemotherapeutics eliminate transformed cells through the induction of replication stress and DNA damage [1,2,3]. Hydroxyurea is used as cytoreductive therapy for leukemic disorders and brain tumors [4,5,6,7]. This drug specifically inhibits ribonucleotide reductase (RNR) and thereby depletes the cellular dNTP pool [8]. This stalls ongoing DNA replication forks and, due to ongoing DNA helicase activity, single-stranded DNA (ssDNA) stretches accumulate. These are covered and protected by replication protein A (RPA). If replication stress persists and depletes the free RPA pool, ssDNA stretches become DNA double-strand breaks (DSBs). These are positive for histone H2AX phosphorylated at S139 (ɣH2AX) [9,10].

We reported that chronic and acute myeloid and lymphoid leukemic cells showed differential sensitivities to apoptosis induction by hydroxyurea [11]. Chronic myeloid leukemia (CML) cells that are driven by the tyrosine kinase BCR-ABL1 (encoded by the reciprocal chromosomal translocation t(9;22)) [12,13] display particular robustness to apoptosis upon dNTP depletion by hydroxyurea [11]. Despite the long-standing use of hydroxyurea in the clinic [4,5,6,7], molecular details on specific signaling pathways that determine whether leukemic cells are responsive or resistant to pro-apoptotic effects of this drug are still to be identified. The modulation of such mechanisms in hydroxyurea-treated cells could deliver novel and improved treatment options. This might be relevant for CML that cannot be treated with tyrosine kinase inhibitors (TKi), the mainstay therapy for this disease [12,13]. Such patients carry mutations in BCR-ABL1, are intolerant to TKi, or experience tumor recurrence upon discontinuation of TKi therapy [14,15]. Improved therapies are likewise necessary for poor-prognosis acute myeloid leukemia (AML) patients with internal tandem duplication in the FMS-like tyrosine kinase (FLT3-ITD). Such cells are susceptible to specific TKi but become drug-refractory due to secondary, therapy-associated tyrosine kinase mutations in the FLT3 kinase domain (FLT3-TKD) [16,17,18].

Using unbiased proteomics, we could identify proteins that are regulated differentially in leukemic cells with a high or low sensitivity to apoptotic effects of hydroxyurea. These proteins include the serine/threonine kinase cellular RAS-associated factor-1 (RAF1/c-RAF). We can demonstrate that RAF proteins regulate the expression of the anti-apoptotic BCL2 family member BCL-XL to protect CML cells from hydroxyurea-induced cell death. This mechanism consequently offers a previously unidentified vulnerability to inhibitors of BCL-XL, RAF1, and their upstream driver kinase BCR-ABL1. To validate our data across systems, we used AML cells that carry mutant FLT3. FLT3-ITD and RAF1 also sustain BCL-XL expression in such cells to protect them from the pro-apoptotic effects of hydroxyurea. Moreover, our data reveal that leukemic cells with an expression FLT3-TKD are susceptible to apoptosis induction by hydroxyurea and navitoclax.

## 2. Material and Methods

### 2.1. Proteomics

Sample preparation and mass spectrometry were performed as described previously by our group [19].

### 2.2. Cell Lines

We used human BCR-ABL1-positive CML cells (K562, KYO-1), PML-RARα-positive acute promyelocytic leukemia cells (APL, NB4 cells) and NB4 cells overexpressing BCL-XL [11], FLT3-ITD-positive biphenotypic myelomonocytic leukemia cells (MV4-11), interleukin-3 (IL-3)-dependent parental murine pro-B cells (Ba/F3), quizartinib-sensitive Ba/F3 cells with FLT3-ITD (Ba/F3-ITD-598), quizartinib-resistant Ba/F3 cells with FLT3-ITD+D835Y and Ba/F3 FLT3-ITD+N676K cells (FLT3-ITD/tyrosine kinase domain double mutants), Ba/F3 cells with constitutively active STAT5A or a mutant thereof lacking an essential O-GlcNACylation site that is required for full STAT5 activity through enhanced tyrosine phosphorylation (Ba/F3 cS5-T92A) [20]. Cells were cultured in RPMI-1640 (Sigma-Aldrich, Munich, Germany), 10% FCS, 1% penicillin/streptomycin (Thermo Fisher, Gibco, Braunschweig, Germany), and 2% L-glutamine. Cells were confirmed to be free of mycoplasma. K562 and NB4 cells were kindly provided by Manuel Grez (Frankfurt/Main, Germany) and Christian Wichmann (Munich, Germany), MV4-11 and BaF3 cells by Frank-Dietmar Böhmer (Jena, Germany) and Thomas Kindler (Mainz, Germany), and KYO-1 cells by Jörg Hartkamp (Aachen, Germany). Cells were verified by DNA fingerprint at the DSMZ, Braunschweig, Germany. We used no commonly mischaracterized cells.

### 2.3. Drugs and Chemicals

Hydroxyurea was from Sigma-Aldrich, Taufkirchen, Germany; imatinib was from Enzo, Lörrach, Germany; navitoclax/ABT263, LY3009120, quizartinib and Z-VAD-FMK were from Selleckchem, Munich, Germany; BP-1-108 is described in [21]. Hydroxyurea was prepared freshly. Stock solutions in DMSO were stored at −80 °C. Before treatment, all drugs were diluted in PBS, except for navitoclax, which was diluted in culture medium.

### 2.4. Transfection of siRNA

A total of 100 pmol siRNA (nontargeting control siRNA-B/sc-44230 or siRNA targeting BCL-XL/LQ-003499-00-0002 (Dharmacon, Lafayette, CO, USA)) was electroporated into 1 × 10^6^ K562 cells with the Amaxa^®^ Nucleofector^®^ II Device (Lonza, Köln, Germany) using program T-016 and Ingenio kit solution (Mirus, Darmstadt, Germany; 100 µL solution per sample). Cells were incubated for 24 h in 2 mL fresh medium/well. The next day, without any washing steps in between, 2 mL fresh medium was added. After 2 h of adaptation, cells were treated with 1 mM hydroxyurea and harvested 24 h later.

### 2.5. Immunoblot

Immunoblots were performed according to [11]. For each membrane, a housekeeping protein is depicted (HSP90, GAPDH, vinculin), which is always shown underneath the detected proteins of interest. Antibodies were: BCL-XL (#ab32370), GAPDH (#ab128915), WT1 (#ab89901) from Abcam, Cambridge, U.K.; RAF1 (#sc-133), HSP90 (#sc-13119), vinculin (#sc-736), ɣH2AX (#sc-101696) from Santa Cruz, Heidelberg, Germany; cleaved caspase-3 (#cs9661), p-Tyr202/Tyr204-ERK1/ERK2 (#cs9101), ERK1/ERK2 (#cs9102) from Cell Signaling, Leiden, Netherlands; p-Tyr694-STAT5 (#MA5-14973) from Thermo Fisher, Braunschweig, Germany; STAT5 (#610192) from BD Bioscience, Heidelberg, Germany; and PARP1 (#556362) from Pharmingen, Heidelberg, Germany. The protein ladders used were the prestained Scientific^TM^ PageRuler^TM^ (#26617) and the PageRuler^TM^ Plus (#26620) from Thermo Fisher, Braunschweig, Germany.

### 2.6. Flow Cytometry

Changes in *Ψm* (Δ*Ψm*) were measured by DiOC6 (Molecular Probes, Dreieich, Germany) staining [11]. For annexin-V/PI staining, cells were washed in PBS, resuspended in 50 µL 1x annexin-V binding buffer (10× stock: 100 mM HEPES, 1,4 M NaCl, 25 mM CaCl_2_, 1% BSA, pH 7,4). Staining was performed with 2.5 µL annexin V-FITC (Miltenyi Biotec, Bergisch Gladbach, Germany) at room temperature for 15 min in the dark. Before measurement, 10 µL PI (stock solution: 50 µg/mL) were diluted in 430 µL annexin binding buffer and added to the cell suspension. Viable cells are annexin-V-/PI-negative, early apoptotic cells are annexin-V-positive/PI-negative; late apoptotic or necrotic cells are annexin-V-positive/PI positive [11,22]. For cell cycle analysis, cells were washed with PBS, resuspended in 100 µL PBS, and fixed with 2 mL ice-cold 80% ethanol for 1 h up to 1 week at −20 °C. After centrifugation, ethanol was removed, and cells were incubated in 1 µL RNase-A (1 µg/mL) (Sigma-Aldrich, Taufkirchen, Germany) per 333 µL PBS (1 h, room temperature). Cells were stained with 167 µL propidium iodide (PI; Sigma-Aldrich, Taufkirchen, Germany; stock solution: 50 µg/mL). Samples were measured on a FACS Canto II flow cytometer with FACSDiva 7.0 software (BD-Biosciences, Heidelberg Germany; FITC-channel).

### 2.7. Alkaline Comet (Single-Cell Gel Electrophoresis) Assay

K562 cells were seeded at 1.5 × 10^5^ cells/mL. A total of 24 h later, cells were treated with inhibitors and harvested after 24 h. Positive control cells were treated with 200 µM tert-butyl hydroperoxide (t-BOOH) (Sigma-Aldrich, Taufkirchen, Germany) for 30 min. Alkaline comet assay was performed as described in [19]. DNA damage (tail intensity) was evaluated with Comet IV software (Perceptive Imaging, Liverpool, U.K.). Per experiment, at least 50 cells were measured for each condition.

### 2.8. Statistics

Statistical analyses were performed with GraphPad Prism 6. Significance was determined by calculating p-values with *t*-test or two-way ANOVA (* *p* ≤ 0.05; ** *p* ≤ 0.01; *** *p* ≤ 0.001; **** *p* ≤ 0.0001). Statistics that are provided for the outcome of annexin-V/PI experiments list early apoptosis first and then late apoptosis/necrosis.

## 3. Results

### 3.1. Global Identification of Factors That Control Apoptosis in Response to Replication Stress

**Figure 1:**
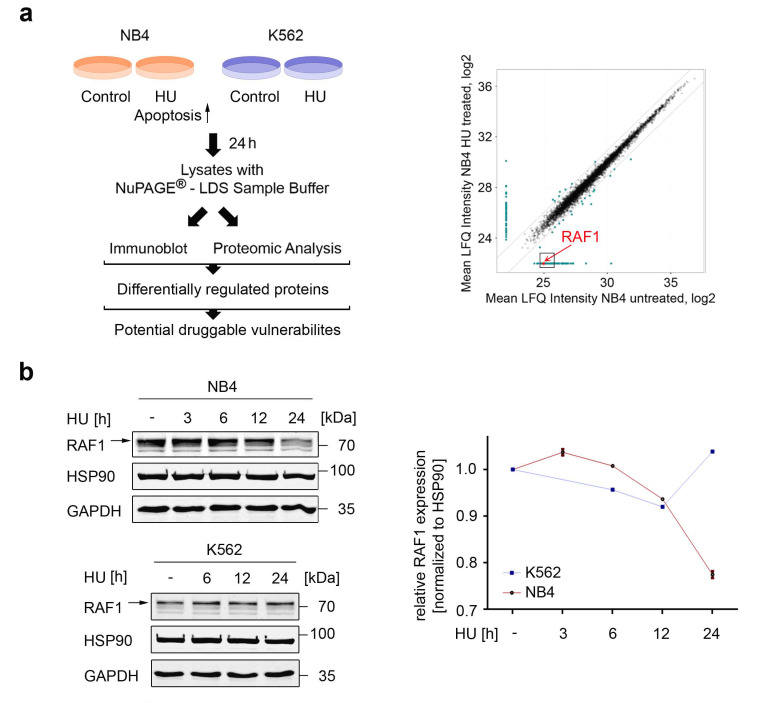
Downregulation of RAF1 in leukemic cells that are sensitive to hydroxyurea. (**a**) Left: Scheme of how NB4 and K562 cells were treated with 0.5 mM hydroxyurea (HU) for 24 h. Aliquots of the cells were taken to perform immunoblot analysis and global proteomics. Right: Global-scale proteomics of NB4 cells after a 12 h treatment with 0.5 mM HU. Scatter plot shows treated versus untreated cells. RAF1 was strongly reduced upon treatment; *n* = 3; *p* = 0.0003. (**b**) Left: Immunoblot of lysates from NB4 cells treated with 0.5 mM HU for 3–24 h and of K562 cells treated with 0.5 mM HU for 6–24 h shows RAF1. Arrows point to the indicated protein. Bands below 70 kDa are non-specific bands (see https://www.proteinatlas.org/ENSG00000132155-RAF1, accessed on 29 April 2021). HSP90 and GAPDH serve as independent loading controls that were applied to the same membrane. Right: Densiometric evaluation of relative RAF1 protein expression in HU-treated NB4 and K562 cells. Values were normalized to HSP90; the untreated control is set as 1; *n* = 2 ± SD.

We set out to identify key regulators of cell fate upon replication stress. We hypothesized that we could identify proteins that render leukemic cells sensitive to apoptosis induction upon replication stress by comparing cells with differential sensitivity to hydroxyurea. Moreover, we reasoned that such proteins might be druggable targets and hence, an Achilles heel of tumor cells that survive conditions of replication stress. To achieve these goals, we compared two cellular systems that show very high (NB4 APL cells) or very low (K562 CML cells) sensitivity to hydroxyurea-induced apoptosis [11]. We incubated them with a clinically achievable dose of 0.5 mM hydroxyurea [7] for 24 h and analyzed cell lysates by mass spectrometry (Figure 1a). Proteomics showed that hydroxyurea decreased the levels of RAF1 in NB4 cells but not in K562 cells (Figure 1a, Supplementary Figure S1a).

To control this experiment, we analyzed the stability of the DNA repair protein PARP1 and the transcription factor WT1. Both proteins are processed when hydroxyurea-treated cells undergo apoptosis [11]. PARP1 was cleaved, and WT1 became decreased in NB4 cells but not in K562 cells that were treated with hydroxyurea (Figure S1b). This was linked to a time- and dose-dependent activation of the apoptosis executioner enzyme caspase-3 in hydroxyurea-treated NB4 cells (Figure S1c). Consistent herewith and with our previous data [11], the pan-caspase inhibitor Z-VAD-FMK blunted apoptosis induction by hydroxyurea in NB4 cells. The hydroxyurea-induced processing of PARP1, the cognate activation of caspase-3 [23], and the accumulation of ɣH2AX as a sign of DNA replication stress/DNA damage [10] were attenuated by Z-VAD-FMK (Figure S1d). Flow cytometry for phosphatidylserine on the cell surface, a marker for early apoptosis, and for the accumulation of PI, a marker of loss of cell membrane integrity, which occurs during necrosis or secondary necrosis of apoptotic cells [11,22], confirmed that hydroxyurea caused caspase-dependent apoptosis in K562 cells (Figure S1e).

To exclude that hydroxyurea caused a general loss of kinases, we analyzed our proteome data set for the expression of other kinases. We observed that hydroxyurea reduced and increased several kinases with various functions in cells. Of these, RAF1 was reduced most strongly, with a 10^8^-fold reduction factor (Figure S1f).

RAS-RAF signaling to mitogen-activated protein kinases (MAP2K/MEK) and extracellular regulated kinases (ERK) is a core cancer pathway that regulates cell proliferation, survival, tumorigenesis, and chemoresistance [24,25,26,27,28]. Therefore, we analyzed the apparent regulation of RAF1 further. Immunoblot analyses confirmed that hydroxyurea reduced RAF1 in NB4 cells time-dependently, with clear effects becoming apparent after a 12 h exposure to 0.5 mM hydroxyurea (Figure 1b). Coherent with the proteomics data, RAF1 remained stable in K562 cells that were exposed to hydroxyurea for 24 h (Figure 1b).

These data demonstrate that a reduction in RAF1 by hydroxyurea correlates with the sensitivity of APL cells to the pro-apoptotic effects of this drug.

### 3.2. Assessment of the Biological Relevance of RAF1

**Figure 2:**
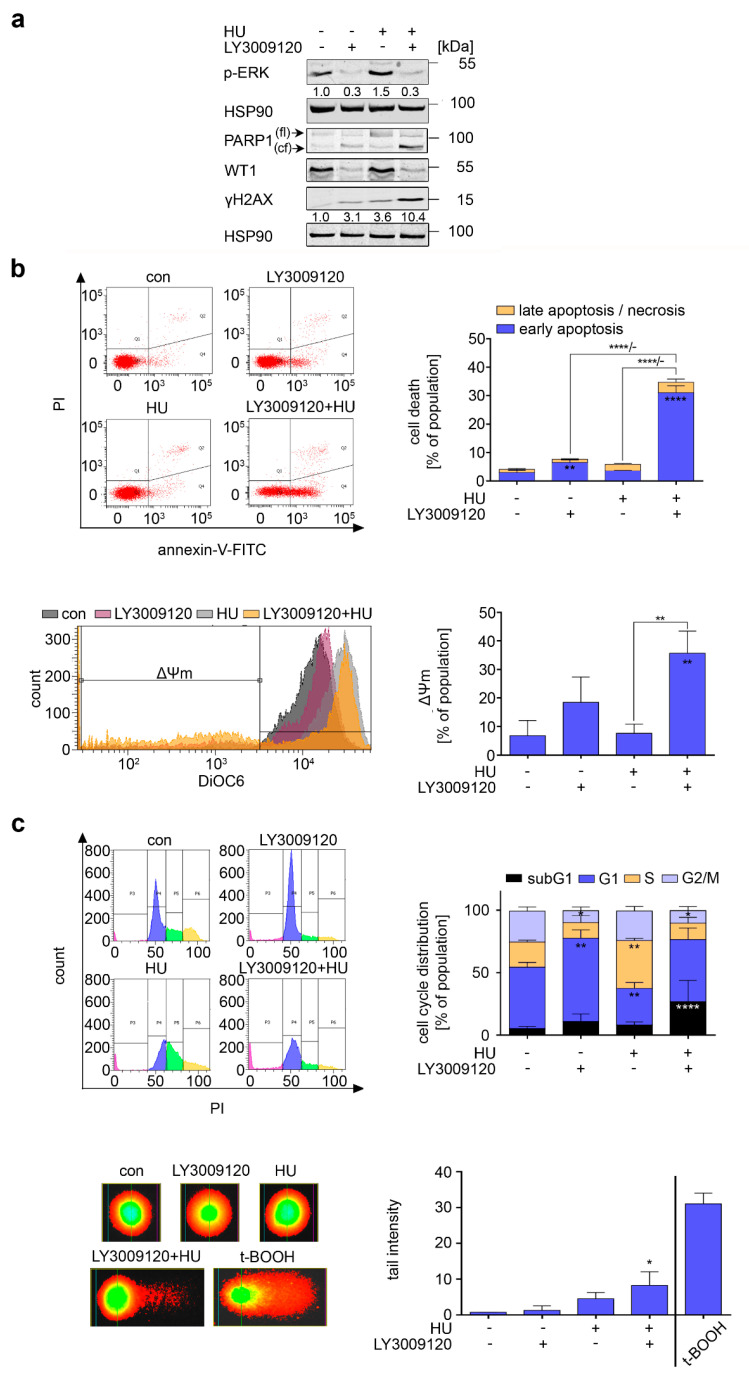
Inhibition of RAF and replication stress induce apoptosis in CML cells. (**a**) Immunoblot of K562 cells treated with 1 µM LY3009120 and 1 mM hydroxyurea (HU) for 24 h show the loss of p-ERK, WT1, and PARP1 and the induction of γH2AX; (fl)—full length, (cf)—cleaved form; HSP90 serves as loading control. Numbers below p-ERK and γH2AX show densiometric analyzes of the protein expression normalized to the loading control; protein levels of untreated cells were defined as 1.0. (**b**) Upper left: Exemplary dot plots of K562 cells exposed to 1 µM LY3009120 and 1 mM HU for 24 h. Cells were stained with annexin-V and PI and measured via flow cytometry for the induction of cell death. Upper right: Calculation of cell death for K562 cells that were treated and stained as stated above; *n* = 3 ± SD; two-way ANOVA; Bonferroni’s multiple comparisons test: ** *p* < 0.01; **** *p* < 0.0001. Lower left: Overlay histogram of DiOC6 stained K562 cells treated as mentioned above. Lower right: DiOC6 stained K562 cells were analyzed for mitochondrial membrane potential (Δ*Ψ*m). Loss of *Ψ*m is an early sign for ongoing cell death; *n* = 3 ± SD; one-way ANOVA; Bonferroni’s multiple comparisons test: ** *p* < 0.01. (**c**) Upper left: Representative histograms of the cell cycle of LY3009120 (1 µM) and HU (1 mM) treated cells (24 h). Upper right: Cells were fixed and stained with PI to analyze their cell cycle distributions; *n* = 3 ± SD; two-way ANOVA; Bonferroni’s multiple comparisons test: * *p* < 0.05; ** *p* < 0.01; **** *p* < 0.0001. Lower left: Alkaline comet assay was performed with K562 cells treated with 1 µM LY3009120 and 1 mM HU for 24 h. Cells treated with 200 µM t-BOOH for 30 min are a positive control for DNA strand breaks. Representative pictures are shown. Lower right: Mean tail intensities for cells treated as mentioned are shown; *n* = 3 ± SD; one-way ANOVA; Bonferroni’s multiple comparisons test: * *p* < 0.05.; more than 50 cells were counted per experiment.

We speculated that the persistence of RAF1 in hydroxyurea-treated K562 cells protected them from cell death. To test this, we applied the third generation RAF inhibitor LY3009120 [29,30] to K562 cells and analyzed whether this drug sensitized them to hydroxyurea-induced killing. The phosphorylation of ERK1/ERK2 at Tyr202 and Tyr204 (hereafter abbreviated as p-ERK) is a readout for RAF activity [29,30]. Hydroxyurea induced a modest but reproducible increase in p-ERK in K562 cells, and LY3009120 suppressed p-ERK in untreated and hydroxyurea-treated K562 cells (Figure 2a).

Immunoblotting revealed that LY3009120 evoked a cleavage of PARP1. Hydroxyurea increased the amount of total PARP1 but not its cleavage in K562 cells (Figure 2a). This is consistent with their resistance to pro-apoptotic effects of hydroxyurea [11]. In K562 cells that were treated with hydroxyurea+LY3009120, the levels of cleaved PARP1 were higher than in the single treatment with LY3009120 (Figure 2a). In addition, LY3009120 and hydroxyurea+LY3009120 caused a near-complete reduction in WT1 (Figure 2a).

Flow cytometry for the staining of cells with annexin-V and PI, markers for early and late apoptosis [11,22], confirmed that hydroxyurea did not significantly cause apoptosis in K562 cells. LY3009120 increased the percentage of early apoptotic cells to 15%, and LY3009120 plus hydroxyurea induced a highly significant induction of 35% early apoptosis after 24 h (Figure 2b).

Analysis of the mitochondrial membrane potential (*ψm*) by flow cytometry, an early event of apoptosis induction [31], corroborated these data. LY3009120 induced mitochondrial injury in up to 19% of K562 cell cultures and hydroxyurea+LY3009120 induced statistically significant levels of mitochondrial injury in 36% of K562 cell cultures (Figure 2b). Hydroxyurea increased DiOC6 staining without a breakdown of *ψm*. These data agree with the observation that cellular stress can lead to increased mitochondrial mass [32].

Flow cytometry analyses for cell cycle progression and apoptotic and necrotic DNA fragmentation further illustrated that LY3009120 significantly increased the percentage of K562 cells in the G1 phase from 50% to 67%. This occurred at the expense of cells in the S and G2/M phases. Hydroxyurea increased the percentage of K562 cells in the S phase significantly from 20% to 39% and reduced G1 phase cells from 49% to 29% (Figure 2c). Coherent with the results from the apoptosis assays, hydroxyurea+LY3009120 significantly increased cytotoxic DNA fragmentation to 27% and reduced the number of cells in G2/M (Figure 2c).

We further analyzed DNA integrity with the alkaline comet assay [33]. Hydroxyurea evoked an increased comet tail intensity of 4.5, indicating ssDNA breaks. Hydroxyurea + LY3009120 augmented this to a significant mean tail intensity of 8.2 (Figure 2c). This correlates with cell death-associated DNA fragmentation (Figure 2c). Consistent herewith, we noted a 10.4-fold accumulation of the replication stress and DNA damage marker ɣH2AX [10] in K562 cells that were incubated with LY3009120 plus hydroxyurea. LY3009120 and hydroxyurea alone caused a 3.1-fold and 3.6-fold increase in ɣH2AX (Figure 2a).

To corroborate our findings, we treated KYO-01 CML cells with LY3009120 and hydroxyurea. In such cells, LY3009120 induced 60% apoptosis, and this number increased to 76% after the addition of hydroxyurea (Figure S2a). LY3009120 caused a significant G1 arrest and hydroxyurea an S phase arrest. The combinatorial treatment stalled cells in the G1 phase, indicating a dominant effect of the RAF inhibitor (Figure S2b).

These results illustrate that RAF activity protects hydroxyurea-treated CML cells from apoptosis.

### 3.3. RAF Promotes Cytoprotective BCL-XL Expression in CML Cells

**Figure 3:**
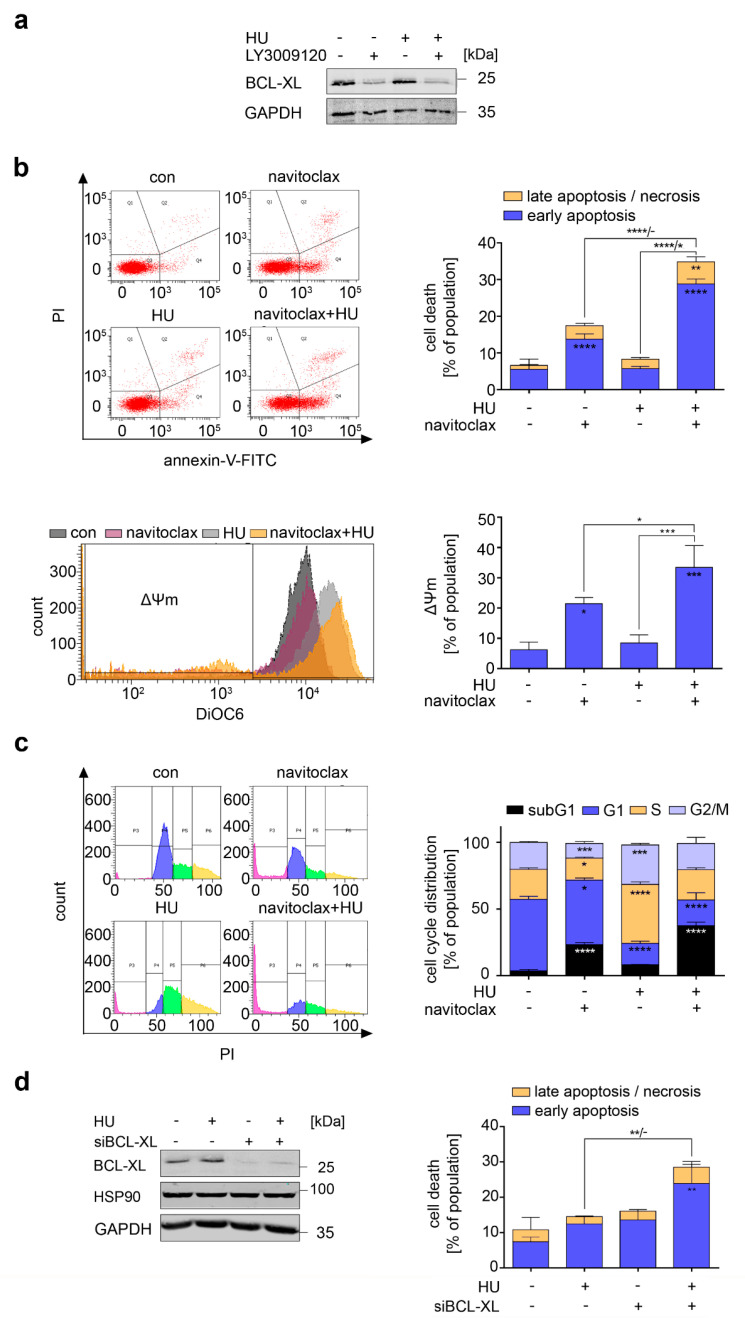
Pro-apoptotic effects of inhibition of BCL-XL are more pronounced in cells with replication stress. (**a**) Immunoblot of K562 cells treated with 1 µM LY3009120 and 1 mM hydroxyurea (HU) for 24 h shows BCL-XL; GAPDH, loading control. (**b**) Upper left: Exemplary dot plots of K562 cells treated with 1 µM navitoclax and 1 mM HU for 24 h. Cells were stained with annexin-V-FITC and PI and analyzed by flow cytometry. Upper right: Cell death calculations of annexin and PI stained K562 cells treated as mentioned before; *n* = 3 ± SD; two-way ANOVA; Bonferroni’s multiple comparisons test: * *p* < 0.05; ** *p* < 0.01; **** *p* < 0.0001. Lower left: Representative overlay histogram of samples treated with 1 µM navitoclax and 1 mM HU for 24 h. Cells were stained with DiOC6 to measure Δ*Ψ*m. Decreased *Ψ*m is a sign of ongoing apoptosis. Lower right: DiOC6 stained cells treated as indicated were analyzed for Δ*Ψ*m; *n* = 3 ± SD; one-way ANOVA; Bonferroni’s multiple comparisons test: * *p* < 0.05; *** *p* < 0.001. (**c**) Left: Cells were treated with 1 µM navitoclax and 1 mM HU for 24 h, fixed and stained with PI. Cell cycle distributions were analyzed by flow cytometry. Shown are representative histograms. Right: Cell cycle distributions of K562 cells treated as mentioned; *n* = 3 ± SD; two-way ANOVA; Bonferroni’s multiple comparisons test: * *p* < 0.05; *** *p* < 0.001; **** *p* < 0.0001. (**d**) K562 cells were transfected with a siRNA against BCL-XL or a non-coding control siRNA. A total of 24 h later, they were treated with 1 mM HU for an additional 24 h. Left: Immunoblot of K562 cells with reduced BCL-XL level through siRNA; HSP90 and GAPDH served as loading controls. Right: Cells were analyzed for the induction of apoptosis via annexin-V and PI staining; two-way ANOVA; Bonferroni’s multiple comparisons test: ** *p* < 0.01.

In transformed B cells, RAF induces the expression of the anti-apoptotic B cell lymphoma family member BCL-XL [28]. This protein protects NB4 cells from the lethal effects of hydroxyurea [11] and promotes the survival of CML cells [34,35]. Therefore, we speculated that LY3009120 reduced BCL-XL and thereby sensitized K562 cells to hydroxyurea. Indeed, LY3009120 strongly downregulated BCL-XL in untreated and hydroxyurea-treated K562 cells (Figure 3a).

These data made us hypothesize that inhibition of BCL-XL could sensitize K562 cells to hydroxyurea. To test this, we inactivated BCL-XL with the clinically tested drug navitoclax [36] in hydroxyurea-treated K562 cells. Flow cytometry revealed that navitoclax plus hydroxyurea caused apoptosis and DNA fragmentation in K562 cells (Figure 3b,c). Upon treatment with navitoclax, 17% of the cells became annexin-V/PI positive, 21% had mitochondrial injury, and 23% showed DNA fragmentation (Figure 3b,c; *p* < 0.05–0.0001).

Hydroxyurea+navitoclax evoked cell death more significantly, with 35% of cells positive for annexin-V/PI, 33% with mitochondrial injury, and 37% with fragmented DNA (Figure 3b,c; *p* < 0.001–0.0001). Furthermore, navitoclax reduced cells in the S and G2/M phases, and hydroxyurea+navitoclax lessened the number of cells in the G1 phase (Figure 3c; *p* < 0.05–0.001).

To genetically corroborate that BCL-XL is a survival protein for hydroxyurea-treated K562 cells, we reduced it with RNAi (Figure 3d), exposed the cells to hydroxyurea, and measured annexin-V/PI by flow cytometry. Consistent with our results for navitoclax, 29% of hydroxyurea-treated K562 cells became annexin-V/PI positive upon a knockdown of BCL-XL (Figure 3d).

These data are not limited to K562 cells. KYO-01 cell cultures were very sensitive to navitoclax, reaching 42% of annexin-V/PI positivity. This amount increased to 50% after the addition of hydroxyurea (Figure S3a). We correspondingly detected significant DNA fragmentation upon the treatment with hydroxyurea+navitoclax (Figure S3b). Furthermore, overexpression of BCL-XL in NB4 cells [11] renders them significantly less sensitive to apoptosis induction by hydroxyurea. Compared to non-transfected NB4 cells, NB4 cells with overexpressed BCL-XL and K562 cells have less mitochondrial injury and become less positive for annexin-V/PI when they are exposed to hydroxyurea (Figure S3c and [11]).

These observations demonstrate that RAF1 and BCL-XL are pharmacological vulnerabilities in hydroxyurea-treated CML cells.

### 3.4. Consequences of BCR-ABL1 Inhibition in CML Cells

**Figure 4:**
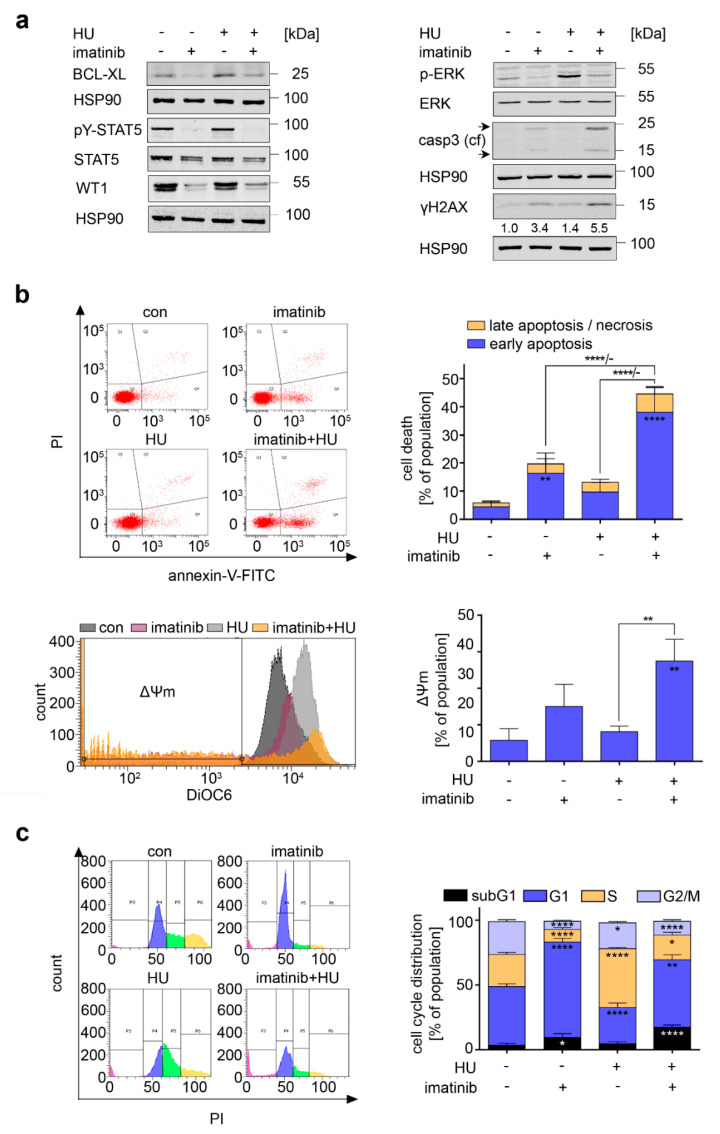
Pro-apoptotic effects of imatinib are more pronounced in cells with replication stress. (**a**) K562 cells were treated with 1 µM imatinib and 1 mM hydroxyurea (HU). Immunoblot analysis shows protein levels of BCL-XL, pY-STAT5, STAT5, WT1, p-ERK, ERK, cleaved caspase-3 (casp3 (cf), caspase-3—cleaved form) and ɣH2AX.; HSP90 serves as loading control. Arrows indicate the active caspase-3 cleavage products. Numbers below ɣH2AX blots are densiometric values of the band intensities normalized to the loading control; protein levels of untreated cells are defined as 1.0. (**b**) Upper left: Representative dot plots of K562 cells treated with 1 µM imatinib and 1 mM HU for 24 h. Cells were stained with annexin and PI. Upper right: The induction of apoptosis and necrosis was measured for cells treated as stated before; *n* = 3 ± SD; two-way ANOVA; Bonferroni’s multiple comparisons test: ** *p* < 0.01; **** *p* < 0.0001. Lower left: Such cells were stained with DiOC6 to determine the Δ*Ψ*m. Representative overlay histograms are shown. Lower right: Measurement of the loss of *Ψ*m in K562 cells treated as indicated; *n* = 3 ± SD; one-way ANOVA; Bonferroni’s multiple comparisons test: ** *p* < 0.01. (**c**) Left: K562 cells treated as in B) were also fixed and stained with PI to analyze cell cycle distributions. Shown are exemplary histograms. Right: Cell cycle distributions of such cells; *n* = 4 ± SD; two-way ANOVA; Bonferroni’s multiple comparisons test: * *p* < 0.05; ** *p* < 0.01; **** *p* <0.0001.

BCR-ABL1 constitutively activates pro-survival and oncogenic signaling cascades through RAS-RAF-MEK-ERK, the transcription factors WT1 and STAT5, BCL-XL, and other proteins [37,38,39,40]. This may shield K562 cells from pro-apoptotic effects of hydroxyurea and could be a vulnerability to the ABL inhibitor imatinib [13,19,41,42]. Immunoblot revealed that imatinib reduced BCL-XL, pY-STAT5, STAT5, WT1, and p-ERK in untreated and hydroxyurea-treated K562 cells. Caspase-3 became cleaved and ɣH2AX accumulated in imatinib-treated cells, and both were more pronounced in the imatinib+hydroxyurea cotreatment scheme (Figure 4a).

Furthermore, annexin-V/PI-positive cells accumulated to 21% and 45% in imatinib and imatinib+hydroxyurea-treated cells (Figure 4b, *p* < 0.01–0.0001). Mitochondrial membrane destabilization occurred in 15% of imatinib-treated cells and in 27% of cells treated with imatinib+hydroxyurea (Figure 4b, *p* < 0.01). We also noted that imatinib and imatinib+hydroxyurea significantly increased the subG1 fraction (*p* < 0.05–0.001), and imatinib+hydroxyurea reduced cells in the S and G2/M phases (Figure 4c, *p* < 0.05–0.0001).

Imatinib also reduced pY-STAT5, STAT5, WT1, and BCL-XL in KYO-01 cells. This was associated with the cleavage of caspase-3, an accumulation of ɣH2AX, and an increase in annexin-V/PI positive cells. These signs of apoptosis became more evident when imatinib+hydroxyurea was applied (Figure S4a,b). Consistently, hydroxyurea+imatinib induced DNA fragmentation (Figure S4c).

Since STAT5 is a key tumor-relevant target of BCR-ABL1 ([37,43,44] and Figure 4a), we tested if STAT5 is a pro-survival factor for hydroxyurea-treated cells. The STAT5 inhibitor BP-1-108 compromised the survival of K562 cells but did not augment their sensitivity toward hydroxyurea (Figure S5a–c). To extend these tests, we used Ba/F3 cells that express hyperactive STAT5 or the less active STAT5-cS5-T92A. These cells responded like parental Ba/F3 cells to hydroxyurea (Figure S5d). Therefore, we did not consider STAT5 further in our analyses.

To this end, our data reveal that the BCR-ABL1 inhibitor imatinib reduces BCL-XL and p-ERK and sensitizes CML cells to the lethal consequences of hydroxyurea-induced replication stress.

### 3.5. RAF Is a Target in Hydroxyurea-Treated FLT3-ITD-Positive AML Cells

Next, we aimed to verify our data in cells from another type of leukemia. We chose AML cells with mutant FLT3 because, like BCR-ABL1, it induces RAF, BCL-XL, and other pro-survival proteins [27,45,46]. Moreover, FLT3-ITD is an unfavorable prognostic marker that is associated with chemotherapy resistance and relapse [16,17,47].

**Figure 5:**
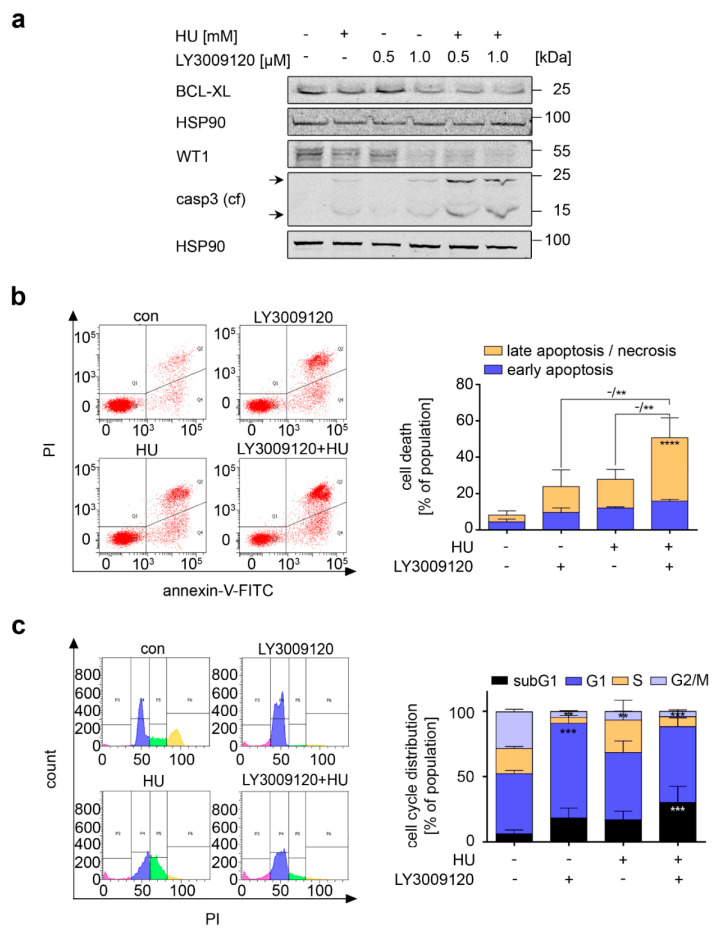
Replication stress and RAF inhibition combine favorably against FLT3-ITD positive AML cells. (**a**) MV4-11 cells were treated with 0.5 mM hydroxyurea (HU) and 0.5 or 1.0 µM LY3009120 for 24 h. Immunoblot shows expression of BCL-XL, WT1, and cleaved caspase-3 (casp3 (cf), caspase 3—cleaved form); HSP90 serves as loading control—note that each membrane was tested for equal loading with HSP90 and shown are the corresponding data for these experiments. (**b**) Left: Representative dot plots of MV4-11 cells treated with 1 µM LY3009120 and 0.5 mM HU for 24 h. Right: Cell death of such MV4-11 cells was measured via annexin-V/PI staining; *n* = 3 ± SD; two-way ANOVA; Bonferroni’s multiple comparisons test: ** *p* < 0.01; **** *p* < 0.0001. (**c**) Left: Exemplary histograms of MV4-11 cells treated with 1 µM LY3009120 and 0.5 mM HU for 24 h. Right: Shown are cell cycle distributions of MV4-11 cells treated as shown above; *n* = 3 ± SD; two-way ANOVA; Bonferroni’s multiple comparisons test: ** *p* < 0.01; *** *p* < 0.001.

We tested whether LY3009120 and hydroxyurea combined favorably against AML cells that carry the hyperactive FLT3-ITD oncogene (MV4-11 cells). LY3009120 and hydroxyurea attenuated BCL-XL and WT1. This was more pronounced in MV4-11 cells that were treated with LY3009120 plus hydroxyurea (Figure 5a). Concomitant with this attenuation of BCL-XL and WT1, we detected cleaved caspase-3 in lysates from cells that we had incubated with hydroxyurea+LY3009120 (Figure 5a).

Flow cytometry for annexin-V/PI demonstrated that hydroxyurea and LY3009120 increased early and late apoptosis in MV4-11 cell cultures. The combined application of these drugs increased the percentage of cells in late apoptosis significantly to 35% from 14% with LY3009120 and 16% with hydroxyurea (Figure 5b).

Congruent herewith, the subG1 fractions increased to 30% in MV4-11 cells that were incubated with hydroxyurea+LY3009120 (Figure 5c, *p* < 0.001). These cytotoxic effects were accompanied by cell cycle alterations. LY3009120 significantly increased the percentage of cells in the G1 phase from 46% to 72% and reduced the cells in the S and G2/M phases. Hydroxyurea significantly reduced the G2/M phase populations. In the combinatorial scheme, a strong reduction in cells in the S phase and the G2/M phase was detectable, and the accumulation of cells in the G1 phase was lost (Figure 5c, *p* < 0.001).

From these results, we conclude that hydroxyurea and LY3009120 combine favorably against AML cells with FLT3-ITD.

### 3.6. Inhibition of FLT3-ITD Sensitizes AML Cells to Hydroxyurea

**Figure 6:**
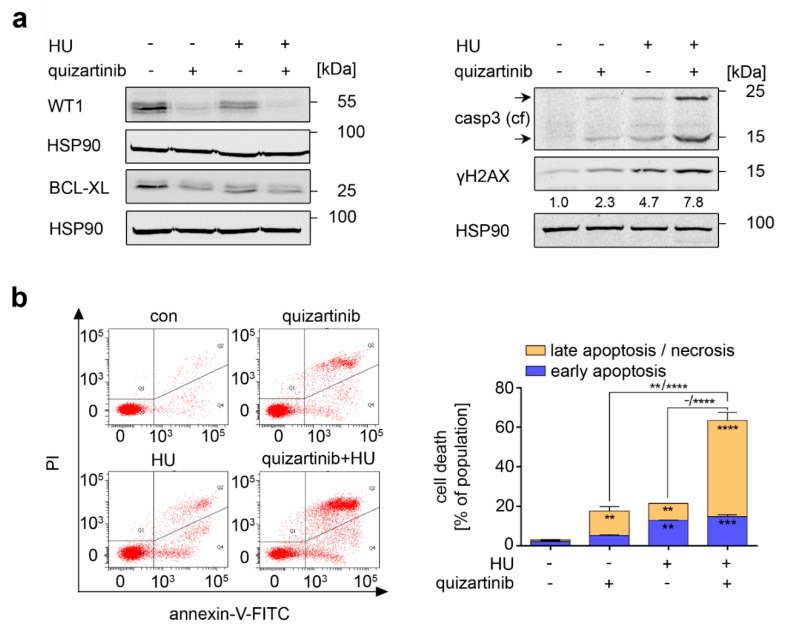

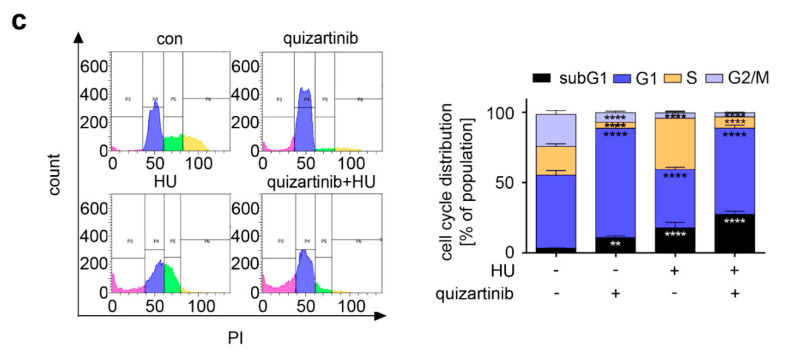
Pro-apoptotic effects of inhibition of FLT3-ITD are more pronounced upon replication stress. (**a**) MV4-11 cells were exposed to 2 nM quizartinib and 0.5 mM hydroxyurea (HU) for 24 h. Immunoblot analysis shows the expression of WT1, BCL-XL; cleaved caspase-3 (casp3 (cf), caspase 3—cleaved form) and γH2AX; HSP90 serves as loading control—note that each membrane was tested for equal loading with HSP90 and shown are the corresponding data for these experiments. Numbers below γH2AX show densiometric analyzes of the protein expression normalized to the loading control; protein levels in untreated cells was defined as 1.0. (**b**) Cells treated as in A) were stained with annexin-V and PI and measured by flow cytometry for the induction of early and late apoptosis and necrosis. Left: Representative dot plots are shown. Right: Cell death calculations of annexin and PI stained MV4-11 cells; *n* = 3 ± SD; two-way ANOVA; Bonferroni’s multiple comparisons test: ** *p* < 0.01; *** *p* < 0.001; **** *p* < 0.0001. (**c**) To analyze the cell cycle distribution, MV4-11 cells treated as indicated were fixed and stained with PI. Left: Exemplary histograms are shown. Right: Cell cycle distribution of these cells; *n* = 3 ± SD; two-way ANOVA; Bonferroni’s multiple comparisons test: ** *p* < 0.01; **** *p* < 0.0001.

The combined application of imatinib and hydroxyurea effectively kills CML cells that are driven by BCR-ABL1 (Figure 4). We investigated whether this equally applies to inhibition of FLT3-ITD with its specific inhibitor quizartinib. Quizartinib reduced WT1 and BCL-XL alone and in combination with hydroxyurea (Figure 6a). Moreover, quizartinib and hydroxyurea led to an accumulation of cleaved caspase-3 and ɣH2AX. The cotreatment potentiated these effects (Figure 6a).

Consistent herewith, hydroxyurea and quizartinib increased the numbers of annexin-V/PI positive MV4-11 cells significantly to 21% and 17%. The combined application of the drugs synergistically potentiated these pro-apoptotic effects to 63% (Figure 6b).

Furthermore, hydroxyurea and quizartinib induced apoptotic DNA fragmentation, and their combined application augmented this (from 18% and 11% to 27%; *p* < 0.0001) (Figure 6c). The single and combined drug treatments reduced the G2/M phase populations significantly. Quizartinib as well as quizartinib plus hydroxyurea decreased the S phase populations to 4% or 8% and induced G1 phase arrest from 52% in untreated cells to 78% in cells treated with quizartinib and 62% in cells treated with both inhibitors (Figure 6c, *p* < 0.001–0.0001).

These data show that hydroxyurea and quizartinib potentiate their pro-apoptotic effects on AML cells.

### 3.7. Navitoclax Combines Favorably with Hydroxyurea against FLT3 Mutant AML Cells

**Figure 7:**
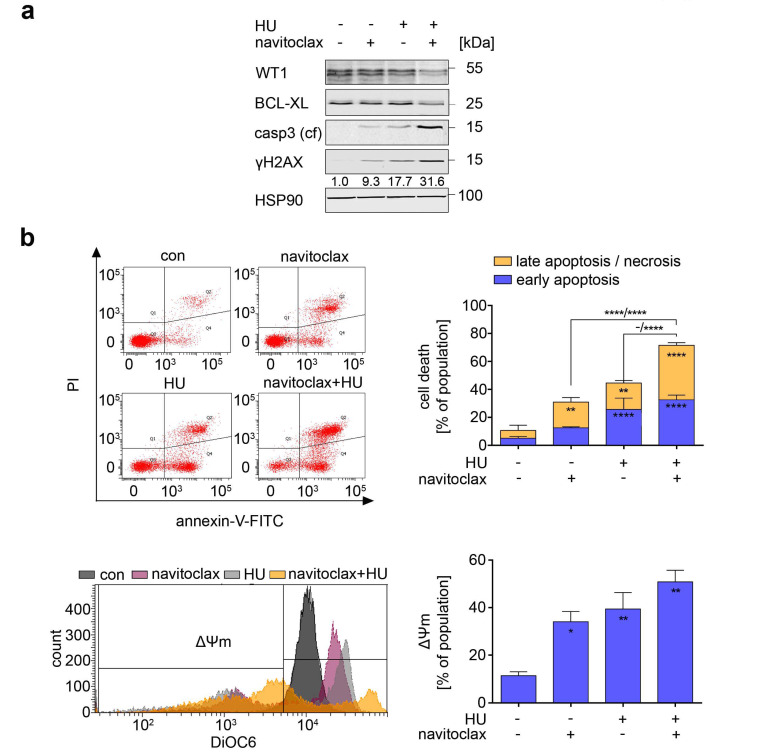

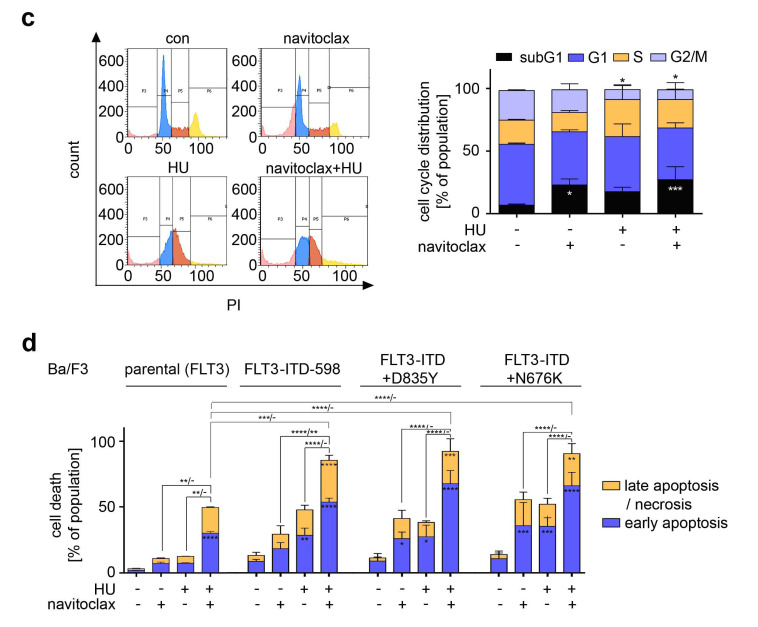
Inhibition of BCL-XL promotes apoptosis upon replication stress in FLT3-ITD positive cells. (**a**) Immunoblot of MV4-11 cells treated with 0.5 µM navitoclax and 0.5 mM hydroxyurea (HU) for 24 h shows protein levels of WT1, BCL-XL, cleaved caspase-3 (casp3 (cf), caspase 3—cleaved form), and γH2AX; HSP90 serves as loading control. Numbers below γH2AX show densiometric analyzes of the protein expression normalized to the loading control; protein level of untreated cells was defined as 1.0. (**b**) Upper left: Exemplary dot plots of MV4-11 cells treated with 0.5 µM navitoclax and 0.5 mM HU for 24 h. Cells were stained with annexin-V-FITC and PI and analyzed by flow cytometry. Upper right: Cell death calculations of annexin and PI stained MV4-11 cells treated as mentioned before; *n* = 3 ± SD; two-way ANOVA; Bonferroni’s multiple comparisons test: ** *p* < 0.01; **** *p* < 0.0001. Lower left: Representative overlay histogram of samples treated with 0.5 µM navitoclax and 0.5 mM HU for 24 h. Cells were stained with DiOC6 to measure Δ*Ψ*m. Decreased *Ψ*m is a sign of ongoing apoptosis. Lower right: DiOC6 stained cells treated as indicated were analyzed for Δ*Ψ*m; *n* = 3 ± SD; one-way ANOVA; Bonferroni’s multiple comparisons test: * *p* < 0.05; ** *p* < 0.01. (**c**) Cells were treated with 0.5 µM navitoclax and 0.5 mM HU for 24 h, fixed and stained with PI. Left: Cell cycle distributions were analyzed by flow cytometry. Shown are representative histograms. Right: Cell cycle distributions of such cells; *n* = 3 ± SD; two-way ANOVA; Bonferroni’s multiple comparisons test: * *p* < 0.05; *** *p* < 0.001. (**d**) Ba/F3 cells were treated with 0.5 µM navitoclax and 0.5 mM HU for 24. Cells were stained with annexin-V-FITC and PI and analyzed by flow cytometry; *n* = 3–4 ± SD; two-way ANOVA; Bonferroni’s multiple comparisons test: * *p* < 0.05; ** *p* < 0.01; *** *p* < 0.001; **** *p* < 0.0001.

LY3009120 and hydroxyurea as well as quizartinib plus hydroxyurea induced apoptosis and reduced BCL-XL in MV4-11 cells (Figure 5 and Figure 6). To test the functional relevance of BCL-XL in these cells, we applied navitoclax with hydroxyurea. Navitoclax and hydroxyurea caused an accumulation of cleaved caspase-3 and ɣH2AX. Their combined application potentiated these effects (Figure 7a).

In agreement herewith, hydroxyurea+navitoclax induced 71% annexin-V/PI positive cells and 51% cells with mitochondrial injury in MV4-11 cultures (Figure 7b; *p* < 0.01–0.00001). Moreover, navitoclax induced DNA fragmentation in 23% of these cell populations, and this increased to 27% upon the addition of hydroxyurea (Figure 7c; *p* < 0.05–0.001).

FLT3-TKD mutants drastically decrease the sensitivity of FLT3 to TKi [16,17,47]. Due to this unresolved clinical issue, we tested navitoclax and hydroxyurea on Ba/F3 leukemia cells with FLT3-ITD (FLT3 with internal tandem duplication at 598) and therapy-associated FLT3-ITD/TKD mutants (FLT3-ITD+D835Y and FLT3-ITD+N676K). Navitoclax induced apoptosis of Ba/F3 cells expressing FLT3-ITD+D835Y and FLT3-ITD+N676K (*p* < 0.05–0.001). Hydroxyurea was effective against Ba/F3 cells with each of the FLT3-ITD isoforms (*p* < 0.05–0.001). In the three Ba/F3 cell types, hydroxyurea+navitoclax evoked over 80% apoptosis (Figure 7d; *p* < 0.01–0.0001). IL-3-dependent Ba/F3 cells with wild-type FLT3 were less susceptible to this drug combination and only had a significant increase in early apoptosis when compared to the single treatments (Figure 7d).

These results illustrate that pharmacological targeting of BCL-XL and hydroxyurea kills FLT3-mutated AML cells, irrespective of whether the cells carry FLT3-ITD or FLT3-TKD.

## 4. Discussion

The induction of replication stress and DNA damage is a mainstay of chemotherapy [1,48,49]. Therefore, it is very important to identify the parameters that determine whether a replication stress program turns into a cytotoxic program. While hydroxyurea hardly evokes cell death in BCR-ABL1-positive CML cells, PML-RARα-positive APL cells and FLT3-ITD-positive AML cells are more susceptible to hydroxyurea-induced apoptosis. This robustness cannot be explained by a lack of replication stress induction. All CML cells, APL, and AML cells that we have tested accumulated typical markers of replication stress upon dNTP depletion, such as ɣH2AX and S phase arrest ([11] and this work). Our data elucidate that BCR-ABL1, FLT3-ITD, RAF1, and BCL-XL suppress apoptosis induction in hydroxyurea-treated CML and AML cells. RAF1 and BCL-XL are known to be activated by the kinases BCR-ABL1 and FLT3-ITD. Accordingly, LY3009120, navitoclax, imatinib, and quizartinib combine significantly with hydroxyurea against leukemic cells with BCR-ABL1 and FLT3-ITD, and these drugs break the resistance of CML cells to hydroxyurea-induced apoptosis.

Unbiased proteomics drew our attention to RAF1, which is important for the growth of leukemic cells [50]. We demonstrate that RAF activity supports the expression of BCL-XL and that BCL-XL is a druggable survival factor in hydroxyurea-treated AML and CML cells. The RAS-RAF-dependent mitogen-activated kinase signaling pathway is also a target in CML cells with point mutants of BCR-ABL1 [25], and hydroxyurea kills CML cells with the imatinib-resistant BCR-ABL1 T315I more effectively than cells with BCR-ABL1 [7]. We show that hydroxyurea and inhibitors of the RAF-BCL-XL signaling node combine favorably against leukemic cells. This finding can be explained by a persistence of RAF1 and BCL-XL in hydroxyurea-treated cells. Moreover, hydroxyurea attenuates RAF1 levels in a time-delayed manner in some cells, whereas an inhibitor of RAF1 immediately tones down RAF1 signaling cascades.

We currently do not know the mechanism for the loss of RAF1 in NB4 APL cells. Our major aim was to solve how the resistance of K562 cells toward hydroxyurea can be broken pharmacologically. Solely due to their highly different susceptibility to hydroxyurea-induced apoptosis, we analyzed the sensitive NB4 APL cells side-by-side with the robust K562 CML cells. As we noted a loss of RAF1 in NB4 cells that were treated with hydroxyurea but not in hydroxyurea-treated K562 cells, we hypothesized that RAF1 protected K562 cells from cytotoxic effects of hydroxyurea. Accordingly, we focused on CML cells and then turned to AML cells with FLT3-ITD, which are clinically challenging [16,17,47]. In contrast to this, APL is mostly curable [51]. Additional work is necessary to decipher how hydroxyurea attenuates RAF1 in certain leukemic cells.

Further research may find that other types of leukemia are susceptible to pharmacological inhibition of RAF-dependent BCL-XL expression. An example could be atypical CML cells lacking the BCR-ABL1 fusion protein [52]. We disclose that hydroxyurea+navitoclax causes apoptosis of leukemic cells with FLT3-TKD mutants. This is important regarding that such mutants disable therapy with the FLT3-specific TKi quizartinib (vulnerable to D835 substitutions [53]) and the broad-range TKi midostaurin (vulnerable to N676K [54]), which is FDA-approved for FLT3-mutant AML [16,17].

Our results are consistent with the finding that combinations of LY3009120 and the BCL2 inhibitor venetoclax efficiently kill AML cells with FLT3 and FLT3-ITD. In cells lacking mutant FLT3, LY3009120 reduced the anti-apoptotic MCL1 protein but not BCL2 [50]. This shows that additional BCL2 family proteins are regulated by RAF and determine survival upon replication stress. Moreover, RAF inhibition can antagonize pro-survival functions of bone marrow mesenchymal cells for AML cells that are treated with the anti-metabolite cytarabine [30]. This treatment kills AML cells without being toxic to healthy bone marrow cells [50]. RAF inhibitors may allow a dose reduction in chemotherapeutics, and this would attenuate side effects on normal dividing cells. Importantly, such an approach is not restricted to leukemic cells with RAS-RAF mutations, which hardly occur in AML and CML cells [7,55]. In this context, it equally has to be considered that the third generation RAF inhibitor LY3009120 inhibits monomers and dimers of all RAF family members and does not induce a paradoxical hyperactivation of wild-type RAF that occurs with more specific RAF inhibitors [30].

Our notion that the sensitivity of CML cells to hydroxyurea is independent of STAT5 agrees with previous results that could not show a linkage between STAT5 activity and the sensitivity of CML cells to hydroxyurea [43]. This does not exclude that STAT5 modulates the susceptibility of leukemic cells toward other chemotherapies. For example, STAT5 promotes the survival of AML and CML cells that are treated with cytarabine [44]. Such differences in the dependency on STAT5 could rely on the different modes of drug actions. While hydroxyurea depletes the dNTP pool and stalls DNA synthesis, without a direct effect on DNA [4,5,6,7], cytarabine is metabolized and becomes incorporated into nascent DNA [56]. Further work is required to delineate the drug-induced DNA damage and repair pathways that STAT5 regulates.

We see that inhibition of BCR-ABL1, FLT3-ITD, or RAF1 causes cell cycle arrest in the G1 phase and that hydroxyurea causes S phase arrest. In contrast to TKi and hydroxyurea, navitoclax has no impact on cell cycle regulation, which is coherent with the expectation that inactivation of BCL-XL and further BCL2 proteins by this drug does not alter cell cycle progression [36]. Nonetheless, administration of navitoclax to hydroxyurea-treated cells was linked to a reduction in the S and G2/M phase cell populations. This suggests that these cell cycle phases are most susceptible to BCL-XL inhibition upon dNTP depletion by hydroxyurea.

The cell cycle arrests that are induced by the kinase inhibitors and hydroxyurea are associated with an accumulation of ɣH2AX. Flow cytometry and comet assays demonstrate that this is not linked to a significant increase in DNA damage. Hence, the induction of ɣH2AX by these drugs is a marker for cell cycle arrest and replication fork stalling. Combinations of the kinase inhibitors and hydroxyurea increase ssDNA breaks and DSBs significantly. It will be interesting to see which of the processes that stabilize and repair DNA during the hydroxyurea-induced S phase arrest [57,58,59] are disrupted by the kinase inhibitors. Such data can provide insights into how the hydroxyurea-induced replication stress turns into lethal DNA damage. Tests are also underway to see whether combinations of kinase inhibitors and new RNR inhibitors, such as the clinically tested COH29 [60], increase DNA damage and apoptosis of leukemic cells.

Imatinib is a gold standard for the treatment of CML cells, and hydroxyurea can be given as cytoreductive therapy [19,41,42]. Imatinib plus hydroxyurea may achieve better responses in patients that are not successfully treated with imatinib and other TKi. Remarkably, hydroxyurea kills CML cells with the imatinib-resistant BCR-ABL1 T351I mutant, and this can be potentiated with the broad-range TKi ponatinib [7]. Thus, hydroxyurea/TKi combinations may eliminate leukemic stem cell clones more effectively, before or upon the advent of drug-resistant mutants. This might allow a discontinuation of TKi therapy. Quizartinib is an FLT3 inhibitor with a narrow range of co-targeted TKs [16,17]. FLT3-mutated AML is still a clinically unmet need, and whether a combined application of FLT3 inhibitors and chemotherapy improves patient survival is tested in clinical trials [16]. According to our preclinical data, it is possible that FLT3 inhibitors and cytoreductive hydroxyurea kill AML cells effectively and that this prevents the selection of cells with secondary FLT3 mutants. Furthermore, targeting RAF1 and BCL-XL as downstream targets of mutant FLT3 and BCR-ABL1 could eliminate leukemic cells irrespective of whether these oncogenic kinases acquired additional mutations during TKi therapy.

## 5. Conclusions

There were over 400,000 prescriptions of hydroxyurea in 2019 in the USA (Hydroxyurea|Sales|Medicare Prescription Data|PharmaCompass.com). Our manuscript is a mechanistic analysis of how hydroxyurea interacts with other drugs against leukemic cells from AML and CML and how the resistance of CML cells toward hydroxyurea can be broken. This work provides insights into novel molecular targets that have the potential for being used in studies involving higher cancer models and prospectively in the clinic. We show for the first time that the RAF1-BCL-XL signaling node protects leukemic cells from cytotoxic effects of replication stress induction by hydroxyurea. This renders RAF1 and BCL-XL as directly and indirectly druggable vulnerabilities to established and currently tested drugs.

## Data Availability

The data are available from the corresponding authors upon request.

## Supplementary Materials

The following are available online at https://www.mdpi.com/article/10.3390/cancers13143464/s1. The file Supplementary Figures and Legends include Figure S1: Effects of hydroxyurea on apoptotic markers in NB4 and K562 cells, Figure S2: RAF Inhibition and hydroxyurea combine favorably against KYO-01 cells, Figure S3: BCL-XL regulates the susceptibility of leukemic cells to hydroxyurea-induced apoptosis, Figure S4: Imatinib and hydroxyurea combine favorably against KYO-01 cells, and Figure S5: Inhibition of STAT5 is cytotoxic for K562 cells. The File Supplementary Original Data shows all original immunoblots with densitometric measurements and original microscopy images and measured tail intensities of the comet assay. The Excel file Supplementary Table shows the results of proteomic analysis of NB4 cells treated with hydroxyurea.
