# Supplementary material for: Oncogenic Kinase Cascades Induce Molecular Mechanisms That Protect Leukemic Cell Models from Lethal Effects of De Novo dNTP Synthesis Inhibition
Source: Cancers (Basel). 2021 Jul 10;13(14):3464. doi: 10.3390/cancers13143464 (PMC8304262; doi:10.3390/cancers13143464)
Supplement: Supplementary file 1 [file cancers-13-03464-s001.zip › Supplementary Figures and Legends.pdf]

cancers

MDPI

Article

Oncogenic Kinase Cascades Induce Molecular Mechanisms that Protect Leukemic Cell Models from Lethal Effects of de novo dNTP Synthesis Inhibition

Miriam Pons, Yanira Zeyn, Stella Zahn, Nisintha Mahendrarajah, Brent D. G. Page, Patrick T. Gunning, Richard Moriggl, Walburgis Brenner, Falk Butter and Oliver H. Krämer

Supplementary Figures and Legends

Pons et al., Figure S1

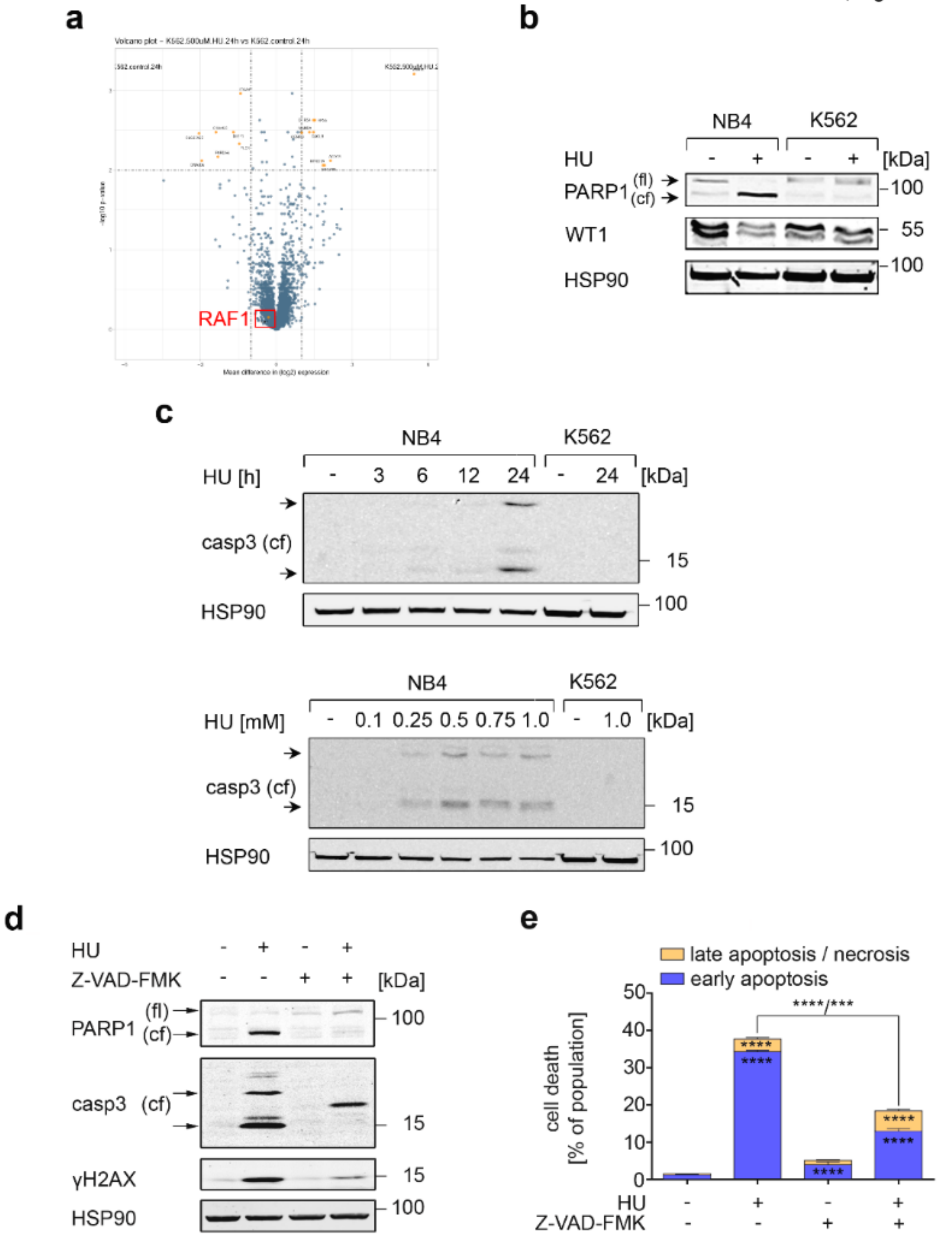

f

NB4 cells			
Gene names	LFQ intensity - NB4 - con	LFQ intensity - NB4 - HU	Trend
Kinases			
**RAF1**	1	**0,00000001**	↓↓↓↓
TP53RK	1	1,15	↑
RBM4B;MST4	1	0,71	↓
PAK2	1	0,37	↓
EIF2AK2	1	0,75	↓
CDK2	1	0,59	↓
CMPK1	1	1,03	↔
PIK3R1	1	2,27	↑
CSK	1	1,17	↑
PXK	1	0,85	↓
MAPK1	1	1,78	↑
SKP1	1	1,30	↑
CDK1	1	0,49	↓
ADK	1	0,84	↓
HK2	1	0,95	↓

red, higher/over 50% increase;
green, lower/over 50% decrease;
yellow, decrease or increase over 25% but below 50%;
↔ change below 5%; rise over 5%; ↓ decrease over 5%

Figure S1. Effects of hydroxyurea on apoptotic markers in NB4 and K562 cells. **a**) K562 cells were treated 5 mM HU for 24 h. Global scale proteomics was performed. Volcano plot shows no significant changes in RAF1 expression. **b**) Immunoblot of lysates from NB4 and K562 cells that were treated with 0.5 mM hydroxyurea (HU) for 24 h shows a loss of the apoptotic markers WT1 and PARP1 in NB4 cells; (fl) - full length, (cf) - cleaved form; HSP90 serves as loading control. **c**) Upper: NB4 and K562 cells were treated with 0.5 mM HU for 3-24 h. Cleaved caspase-3 (casp3 (cf), caspase 3 - cleaved form) was detected by immunoblot; HSP90 serves as loading control. Arrows indicate the active caspase-3 cleavage products. Lower: NB4 and K562 cells were treated with 0.1-1.0 mM HU for 24 h. Active caspase-3 was analyzed by immunoblot; HSP90 serves as loading control; arrows show active caspase-3 cleavage products. **d**) NB4 cells were treated with 50 µM Z-VAD-FMK for 1h before adding 0.5 mM HU for additional 24 h. Immunoblot shows expression of PARP1, caspase 3 and γH2AX; (fl) - full length, (cf) - cleaved form; HSP90 serves as loading control. **e**) Cells treated as in (e) were stained with annexin-V and PI and measured via flow cytometry for the induction of cell death. Calculation of cell death for K562 treated and stained as stated before; $n = 3 \pm$ SD; two-way ANOVA; Bonferroni's multiple comparisons test: ****$p < 0.0001$. **f**) NB4 cells were treated with 0.5 mM HU for 24 h. Global scale proteomics was performed. Heatmap shows how kinase expression changed following the treatment; $n = 3$.

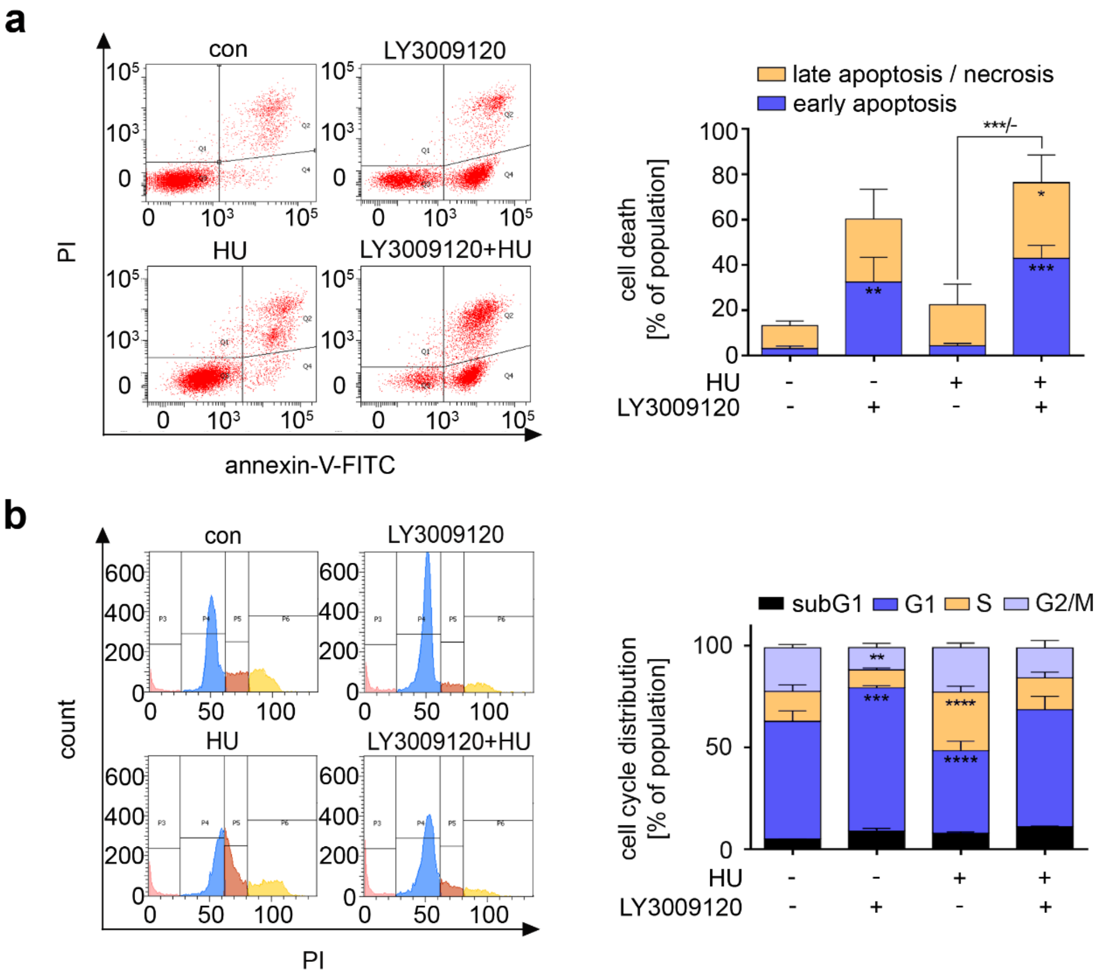

Figure S2. RAF Inhibition and hydroxyurea combine favorably against KYO-01 cells. a) Left: Exemplary dot plots of KYO-01 cells exposed to 1 µM LY3009120 and 1 mM hydroxyurea (HU) for 24 h. Cells were stained with annexin-V and PI and measured via flow cytometry for the induction of cell death. Right: Calculation of cell death for K562 treated and stained as stated before; $n = 3 \pm$ SD; two-way ANOVA; Bonferroni's multiple comparisons test: *$p < 0.05$; **$p < 0.01$; ***$p < 0.001$. b) Left: Representative histograms of the cell cycle of fixed and PI stained KYO-01 cells that were treated with 1 µM LY3009120 and 1 mM HU for 24 h. Right: Cell cycle distributions of such cells; $n = 3 \pm$ SD; two-way ANOVA; Bonferroni's multiple comparisons test: **$p < 0.01$; ***$p < 0.001$.

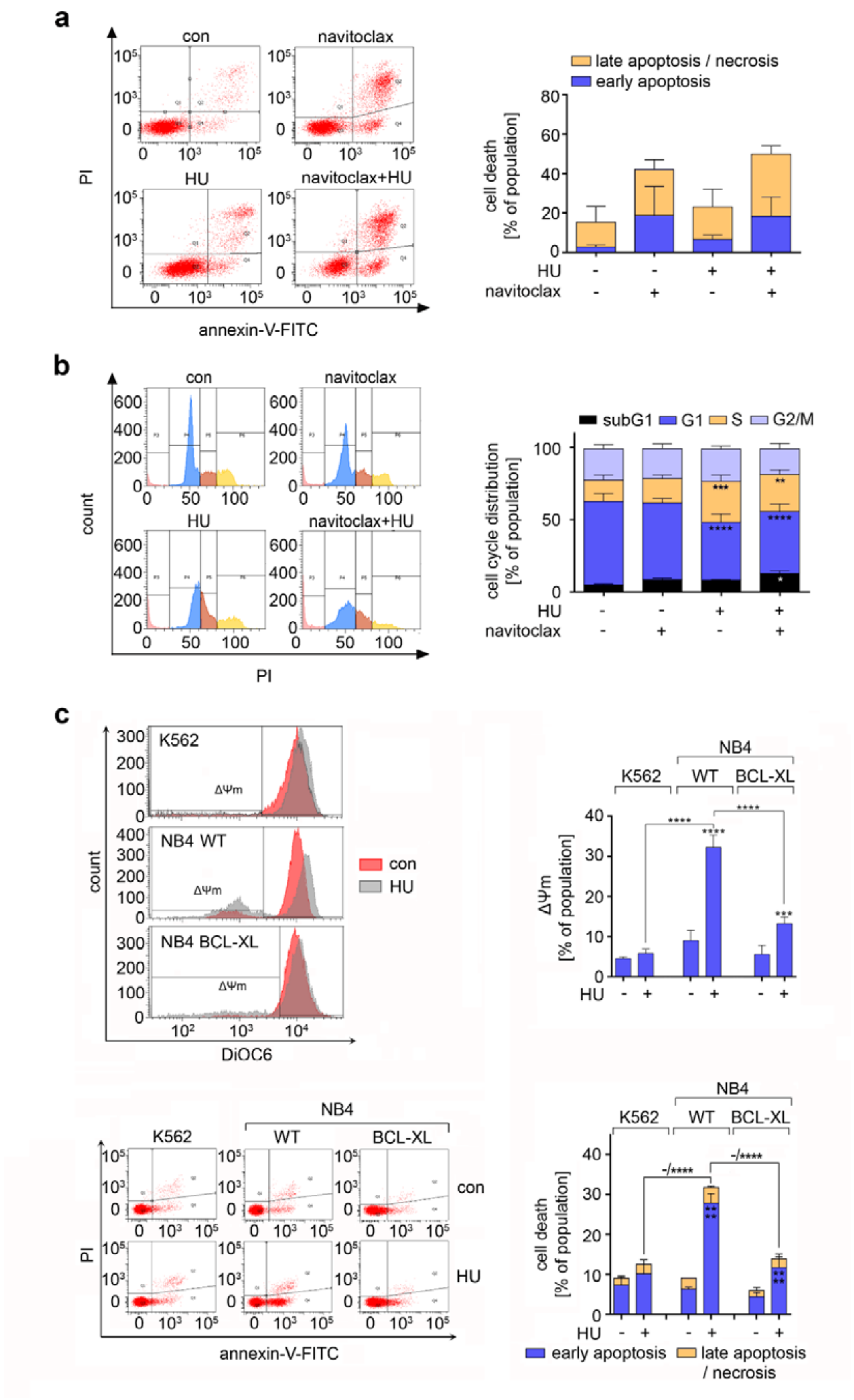

Figure S3. Navitoclax and hydroxyurea efficiently kill KYO-01 cells. **a**) Left: Exemplary dot plots of KYO-01 cells treated with 1 µM navitoclax and 1 mM hydroxyurea (HU) for 24 h. Cells were stained with annexin-V-FITC and PI and analyzed by flow cytometry. Right: Cell death calculations of annexin and PI stained KYO-01 cells treated as mentioned before; n = 3 ± SD; two-way ANOVA; Bonferroni's multiple comparisons test; not significant. (**b**) Such cells were also analyzed for

cell cycle distributions by flow cytometry. Left: Shown are representative histograms. Right: Cell cycle distributions; two-way ANOVA; Bonferroni's multiple comparisons test: ** $p < 0.01$; ** $p < 0.05$; *** $p < 0.001$. **c**) Upper left: Representative overlay histogram of K562 and NB4 wild-type (WT) cells or NB4 cells with BCL-XL overexpression were treated with 0.5 mM HU for 16 h. Cells were stained with DiOC6 to measure ΔΨm. Lower right: DiOC6 stained cells treated as indicted were analyzed for ΔΨm; *n* = 3- ± SD; one-way ANOVA; Bonferroni's multiple comparisons test: *** $p < 0.001$; *** $p < 0.0 =$ 01. Lower left: Exemplary dot plots of K562 an NB4 cells treated with 0.5 mM HU for 16 h. Cells were stained with annexin-V-FITC and PI and analyzed by flow cytometry. Upper right: Cell death calculations of annexin-V/PI-stained cells treated as mentioned before; *n* = 3 ± SD; two-way ANOVA; Bonferroni's multiple comparisons test: **** $p < 0.0001$.

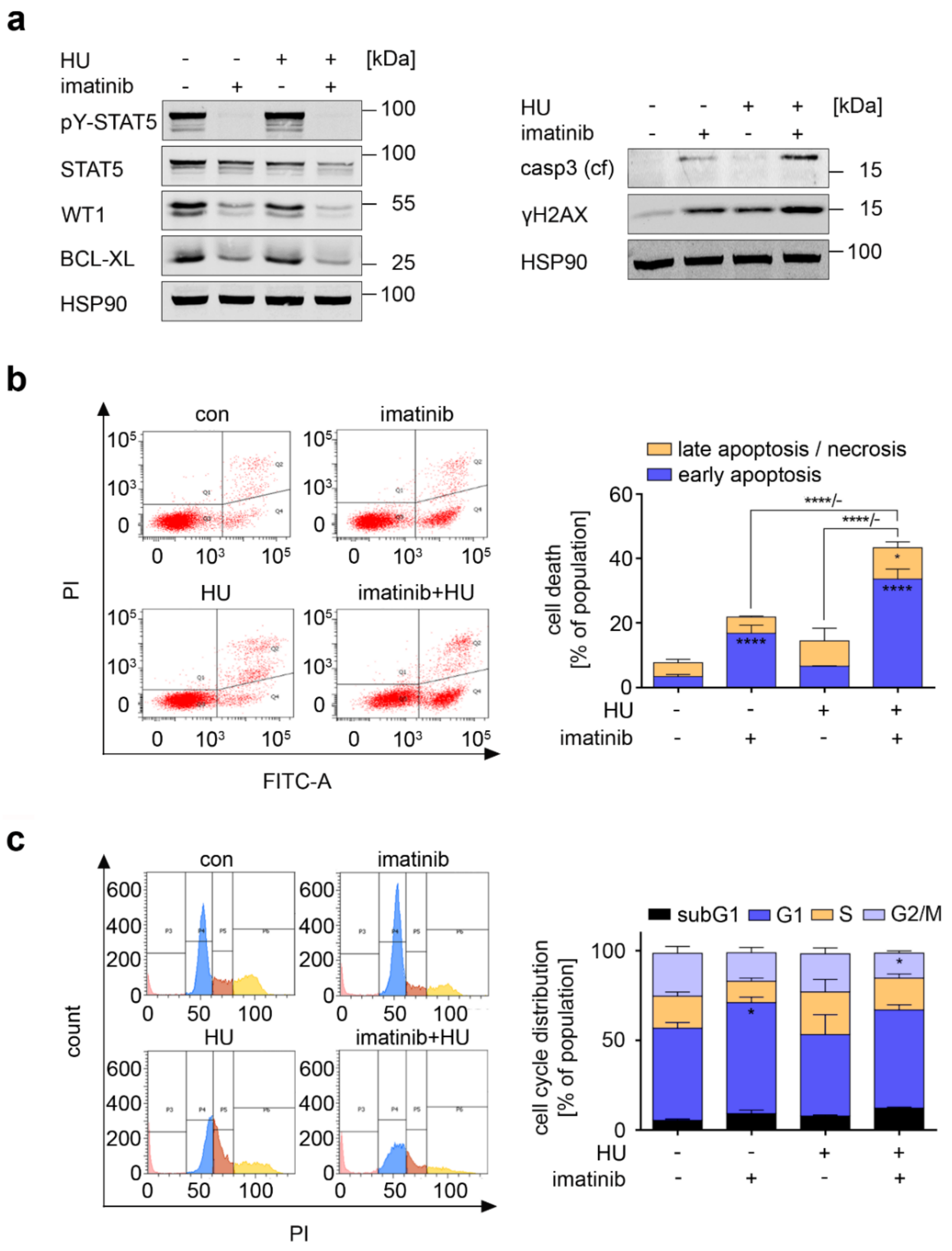

Figure S4. Imatinib and hydroxyurea combine favorably against KYO-01 cells **a)** KYO-01 cells were treated with 1 μM imatinib and 1 mM hydroxyurea (HU). Immunoblot analysis shows pY-STAT5, STAT5, WT1, BCL-XL, cleaved caspase-3 (casp3 (cf), caspase 3 - cleaved form), and γH2AX; HSP90 serves as loading control. **b**) Left: Representative dot plots of KYO-01 cells treated with 1 μM imatinib and 1 mM HU for 24 h. Cells were stained with annexin and PI to determine the induction of cell death. Right: Cell death was measured for cells treated as mentioned before; *n* = 3 ± SD; two-way ANOVA; Bonferroni's multiple comparisons test: * $p < 0.05$; **** $p < 0.0001$. **c**) Cells treated as in B) were fixed and stained with PI to analyze cell cycle distributions. Left: Shown are exemplary histograms. Right: Cell cycle distributions of these cells; *n* = 3 ± SD; two-way ANOVA; Bonferroni's multiple comparisons test: * $p < 0.05$**.**

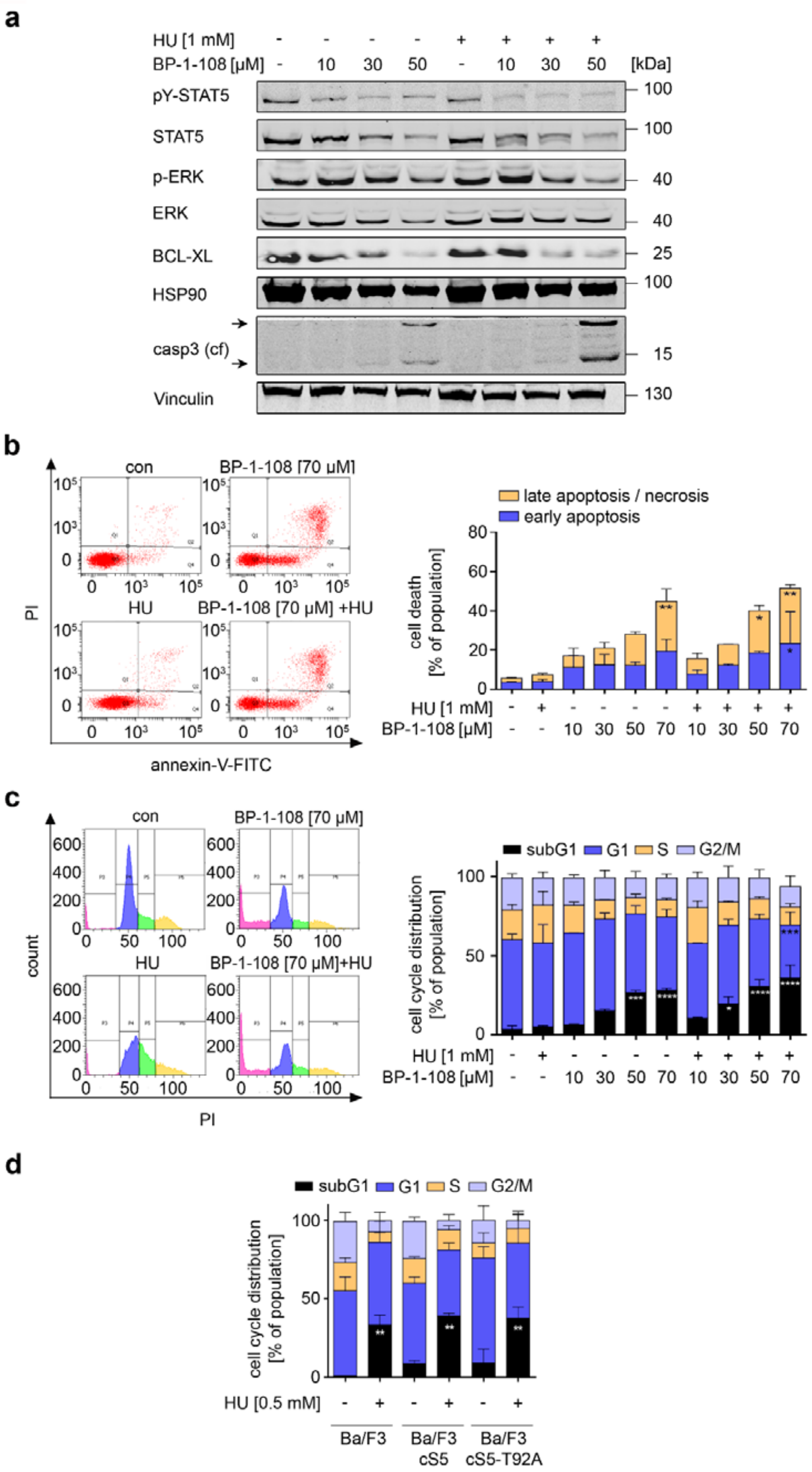

Figure S5. Inhibition of STAT5 is cytotoxic for K562 cells. **a**) Immunoblot of K562 cells treated with 1 mM hydroxyurea (HU) and increasing doses (10-50 μM) of the STAT5 inhibitor BP-1-108 for 24 h. Shown are pY-STAT5, STAT5, *p*-ERK, ERK, BCL-XL, and cleaved caspase-3 (casp3 (cf), caspase 3 - cleaved form); HSP90 and vinculin serve as loading controls. **b**) Left: Exemplary dot plots of K562 cells exposed to 70 μM BP-1-108 and 1 mM HU for 24 h. Cells were stained with annexin-V and PI and measured via flow cytometry for the induction of cell death. Right: Cell death calculations of annexin

and PI stained K562 cells treated with 1 mM HU and 10-70 μM of BP-1-108 for 24 h; $n = 3 \pm$ SD; two-way ANOVA; Bonferroni's multiple comparisons test: *$p < .05$; **$p < 0.01$. **c**) K562 cells treated as in B) were fixed and stained with PI to analyze cell cycle distributions. Left: Shown are exemplary histograms. Right: Cell cycle distributions of K562 cells treated with 1 mM HU and 10-70 μM of BP-1-108 for 24 h; $n = 3 \pm$ SD; two-way ANOVA; Bonferroni's multiple comparisons test: *$p < 0.05$; ***$p < 0.001$; ****$p < 0.0001$. **d**) Parental and cS5 (active) or cS5-T92A (inactive) transfected Ba/F3 cells were treated with 0.5 mM HU for 24 h and analyzed for cell cycle distributions; $n = 2 \pm$ SD; two-way ANOVA; Bonferroni's multiple comparisons test: **$p < 0.01$.
