# Supplementary material for: Oncogenic Kinase Cascades Induce Molecular Mechanisms That Protect Leukemic Cell Models from Lethal Effects of De Novo dNTP Synthesis Inhibition
Source: Cancers (Basel). 2021 Jul 10;13(14):3464. doi: 10.3390/cancers13143464 (PMC8304262; doi:10.3390/cancers13143464)
Supplement: Supplementary file 1 [file cancers-13-03464-s001.zip › Supplementary Original Data.pdf]

Original blots corresponding to Fig. 1B

NB4

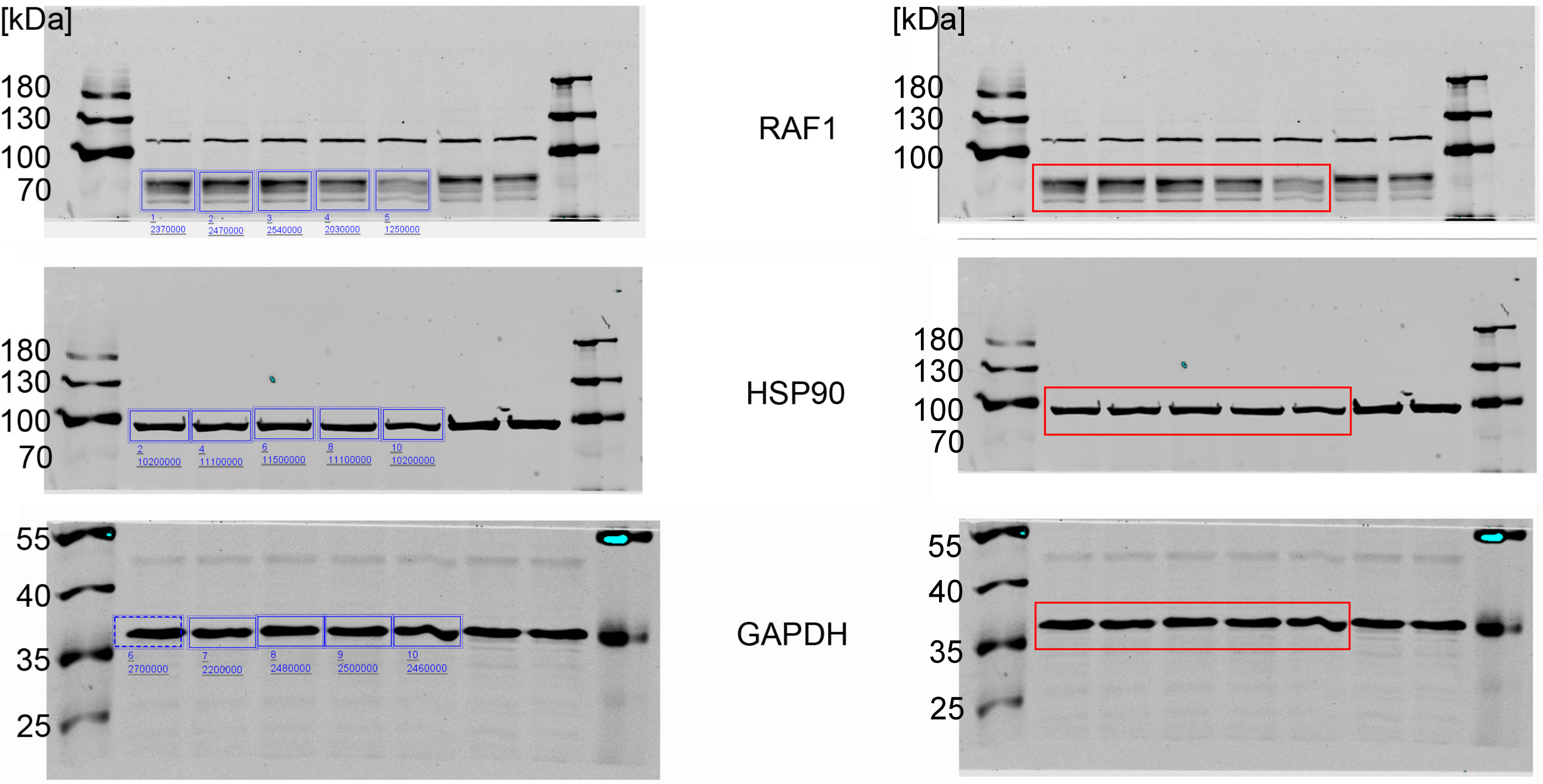

K562

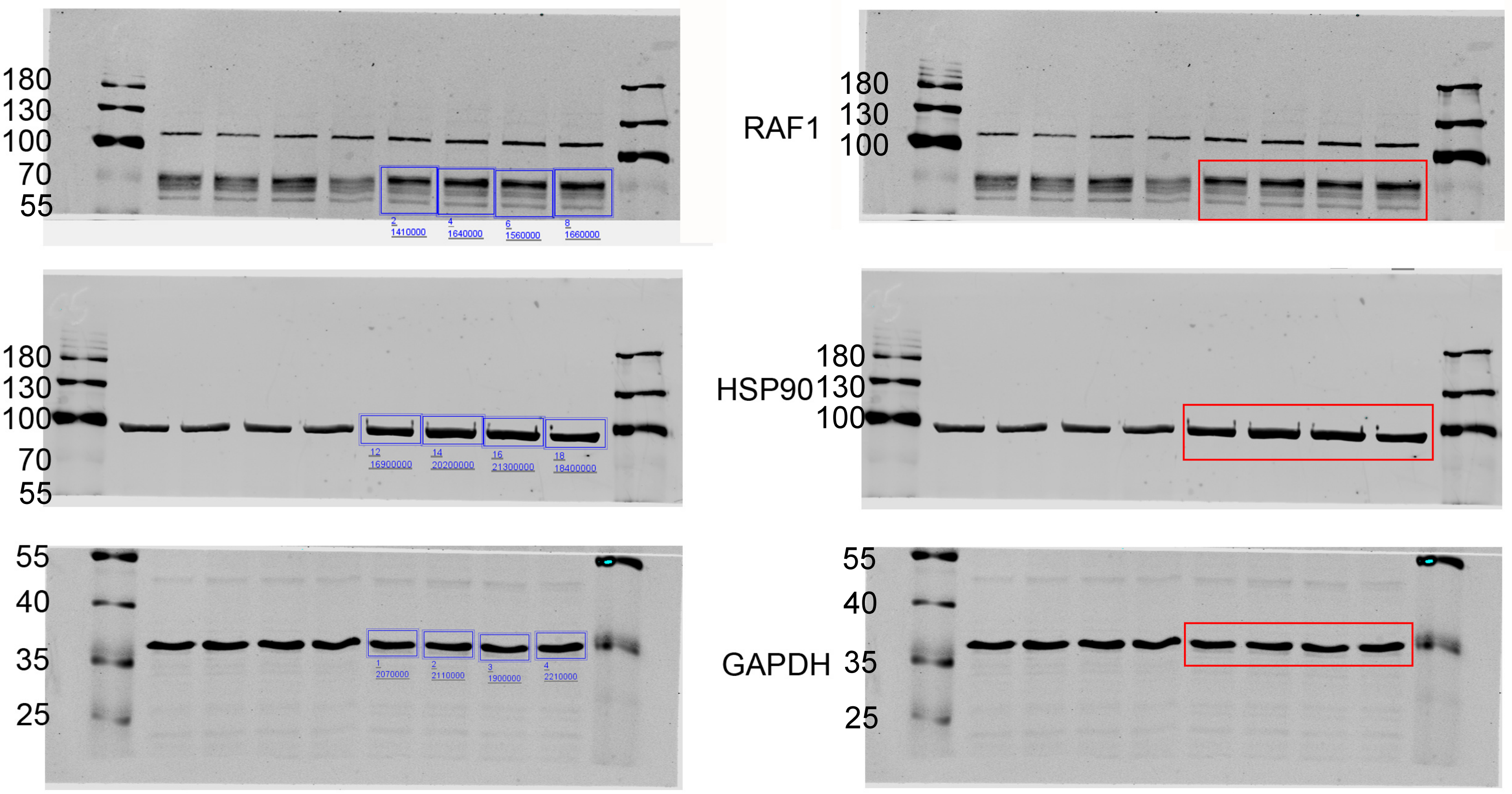

Figure 1B

NB4	RAF1		HSP90		GAPDH	
	Signal	Ratio	Signal	Ratio	Signal	Ratio
con	2370000	1,00	10200000	1,00	2700000	1,00
0.5 mM HU - 3h	2470000	1,04	11100000	1,09	2200000	0,81
0.5 mM HU - 6h	2540000	1,07	11500000	1,13	2480000	0,92
0.5 mM HU - 12h	2030000	0,86	11100000	1,09	2500000	0,93
0.5 mM HU - 24h	1250000	0,53	10200000	1,00	2460000	0,91

K562	RAF1		HSP90		GAPDH	
	Signal	Ratio	Signal	Ratio	Signal	Ratio
con	1410000	1,00	16900000	1,00	2070000	1,00
0.5 mM HU - 6h	1640000	1,16	20200000	1,20	2110000	1,02
0.5 mM HU - 12h	1560000	1,11	21300000	1,26	1900000	0,92
0.5 mM HU - 24h	1660000	1,18	18400000	1,09	2210000	1,07

Original blots corresponding to Fig. 2A

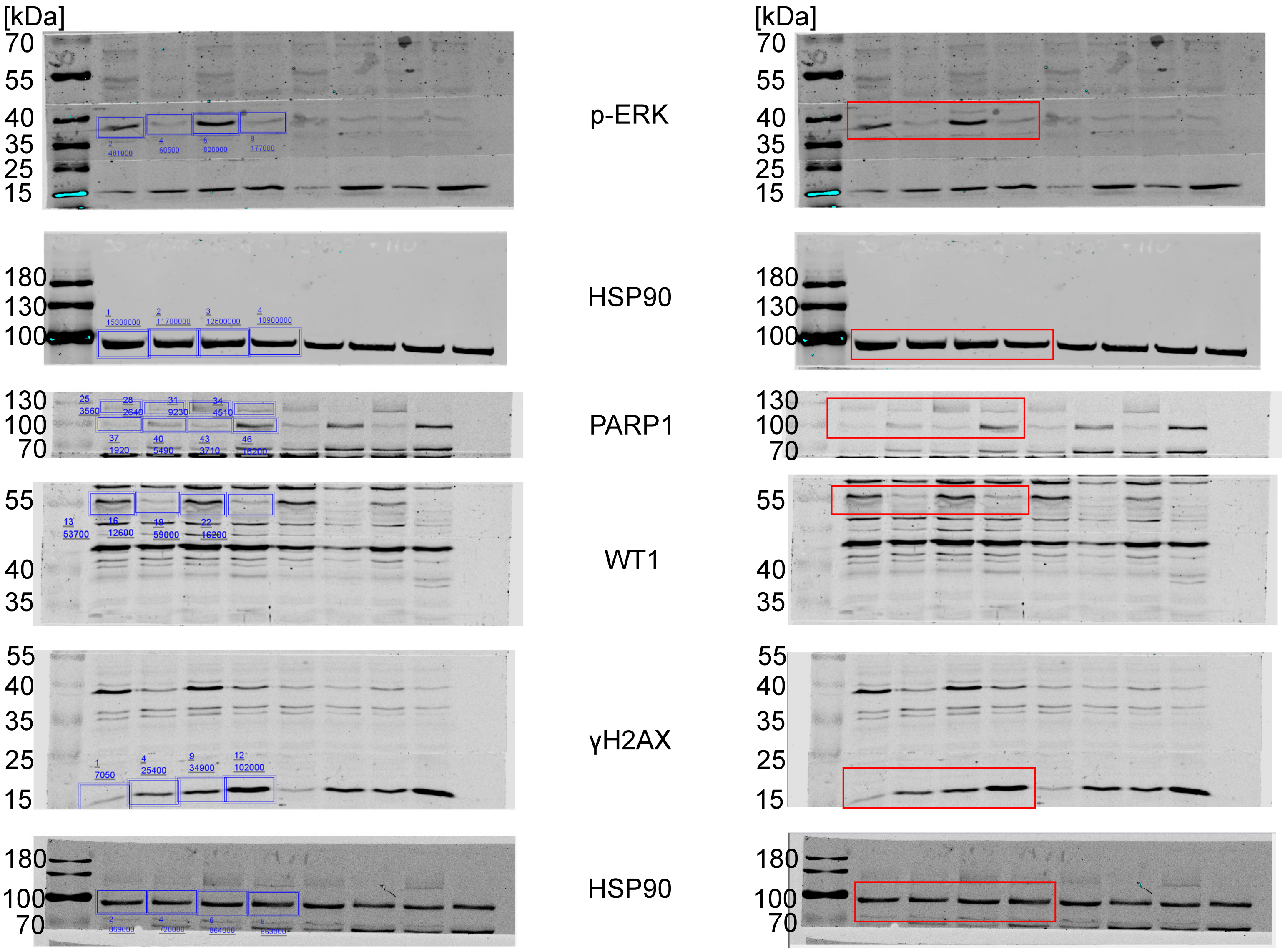

Figure 2A

NB4	p-ERK		HSP90		PARP1 - full lenth		PARP1 - cleaved form		WT1		γH2AX		HSP90	
	Signal	Ratio	Signal	Ratio	Signal	Ratio	Signal	Ratio	Signal	Ratio	Signal	Ratio	Signal	Ratio
con	481000	1,00	15300000	1,00	3560	1,00	1920	1,00	53700	1,00	7050	1,00	869000	1,00
1 µM LY3009120	60500	0,13	11700000	0,76	2640	0,74	5490	2,86	12600	0,23	25400	3,60	720000	0,83
1 mM HU	820000	1,70	12500000	0,82	9230	2,59	3710	1,93	59000	1,10	34900	4,95	864000	0,99
LY3009021 + HU	177000	0,37	10900000	0,71	4510	1,27	16200	8,44	16200	0,30	102000	14,47	863000	0,99

Original microscopy images corresponding to Fig.2c

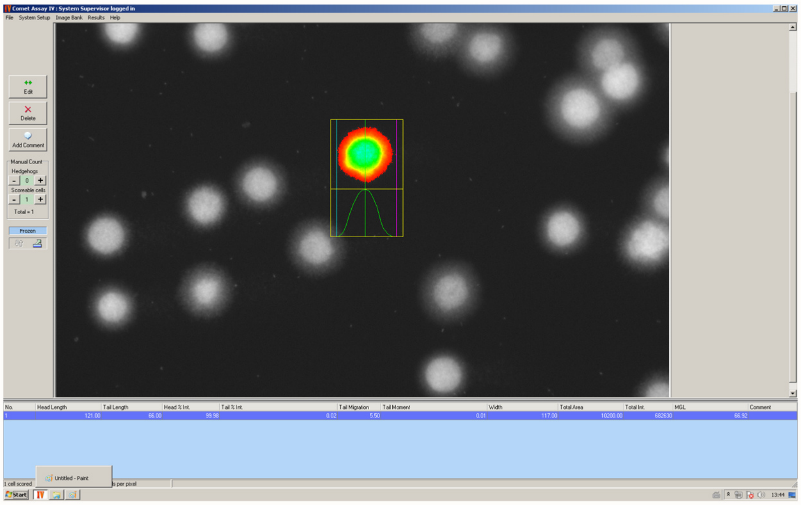

con

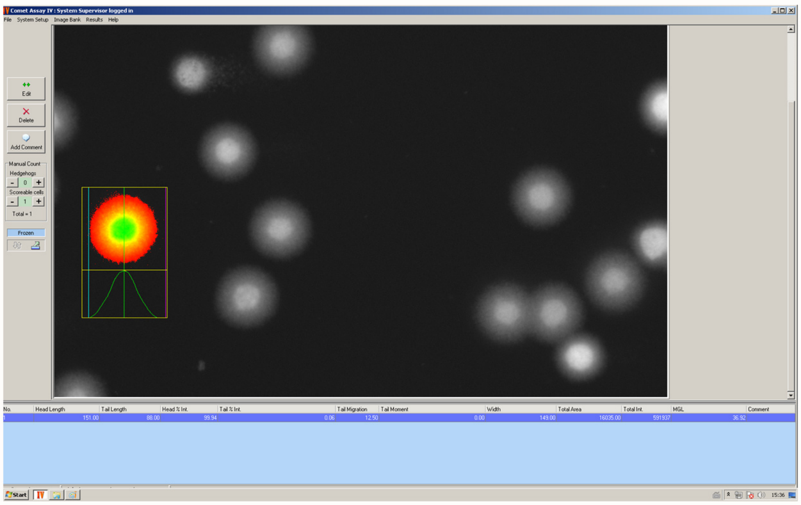

LY3009120

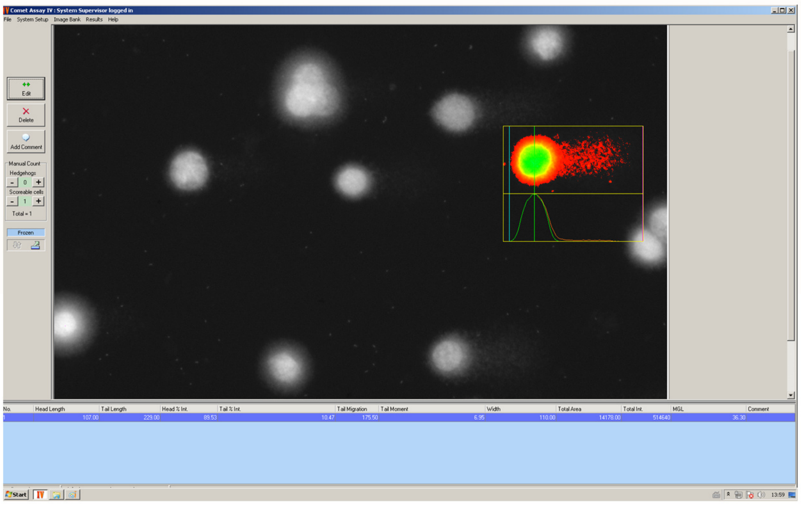

HU

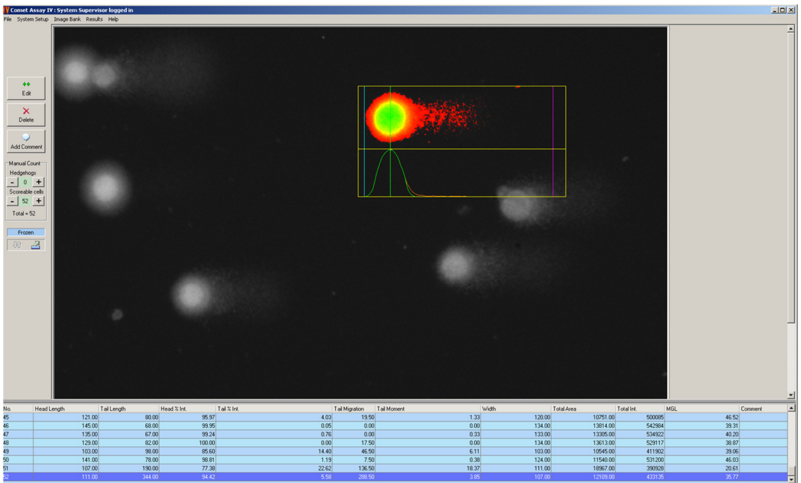

LY3009120+HU

Original measurement of tail intensities corresponding to Fig.2c

Tail Intensities N1

Con	LY	HU	LY+HU	t-BOOH
0,023585	25,71952	11,79604	25,27599	10,46895
0,005212	42,37886	5,876428	0,606415	10,42263
0	7,108165	4,541116	36,13939	53,36935
0,217633	5,109347	11,52234	6,574103	24,91763
0,608757	2,996048	10,90147	2,638994	53,43868
4,190321	0	21,15839	33,14255	40,26614
3,084964	4,000102	4,014333	13,76054	32,98135
0,047653	19,58818	6,355523	0,936477	13,60736
0,015285	2,363267	6,63783	0	42,70174
0,073532	0,833872	10,23058	0,194815	31,48072
0	0,141739	6,142309	12,44292	72,09381
1,480246	4,136098	2,357454	5,686888	35,95858
0,487916	0,253038	2,104191	23,37793	63,09578
0,074483	0,033475	5,1352	0,036689	36,49669
0,353038	1,55542	4,505121	32,01594	52,74082
0,069328	1,640854	12,6201	24,62296	44,7145
0,0169	3,613596	9,656438	44,44215	46,49012
0,001467	0,025315	5,45631	0,333549	32,07999
0,069197	0,051907	9,720165	0,001315	42,87751
0,012656	0,695555	9,395045	0,016067	68,29122
0,143051	0,219534	9,357536	0,441688	53,36391
0	0,019637	6,340476	16,706	27,07962
0	0	3,785896	37,99776	51,38769
0,481293	0,022302	6,273941	0,987668	75,95828
0,897048	0,109096	1,993852	0,050681	67,75103
0,029751	0,149455	7,009875	19,06216	13,16321
0	0,023929	12,27189	24,33422	30,69782
3,388466	0	8,41283	4,353358	32,80296
0,472634	0,001454	2,195441	0,763259	68,86724
0,417351	0,027413	2,267316	24,13969	25,33005
0,026396	0,065968	0,59015	2,399625	46,27753
0,28484	0,004023	11,30115	0,778673	31,07004
0,053518	0,071022	4,694418	0,827115	31,3718
0,003261	3,476541	0,085406	0,608758	50,76527
0,004365	0,087926	11,13306	46,19472	13,77472
3,893963	0,023615	6,197003	3,447084	49,64769
0,626711	0,273923	0,07309	0,198674	23,51777
1,84786	4,296898	4,891735	2,528249	26,8768
0	0,177514	9,435386	3,159314	22,91962
2,387192	3,135945	8,780869	1,350256	24,01087
0,323109	0,550314	3,926767	13,14915	38,68378
0,000537	0,455093	0,807501	27,0035	32,05769
0,061461	0,212418	6,854361	0,011931	28,11788
0,174491	0,16532	1,322534	0,133008	73,67293
0,526104	0,273517	5,45623	12,46667	24,55016
2,996208	0,016079	1,680789	35,4387	73,22026
0,02463	0,258254	5,561208	1,677342	46,88315
0,80317	0,00132	1,203516	23,27207	17,13145
0,073158	0,414244	0,939735	35,94915	22,13437
0,267765	0,14707	8,088621	27,72744	29,02602
0,000653	0,038504	1,584743	2,960009	
	0,550471		25,57232	
	2,122495		1,269903	
	5,112038		19,28136	
	1,356625		26,44073	
			3,93076	
			20,09236	
			19,42576	

Tail Intensities N2

Con	LY	HU	LY+HU	t-BOOH
0,036221	0,010847	10,46712	4,397082	34,66669
0,172845	0,048104	5,292477	0,512713	29,04819
2,323895	0,006975	5,397483	28,3532	38,9421
0,067141	0	7,427472	1,273184	44,32837
0,23114	2,673192	0,007339	0,440784	44,32837
0,593518	0,142224	19,2929	2,839951	50,94172
1,866369	0,081596	7,495452	3,428565	28,63292
3,666598	0	0,742972	11,0935	45,63367
0,31054	0	0,166849	16,79516	30,07371
2,633425	0	1,082423	11,48713	34,7032
2,239209	0	0,454153	2,876717	39,42777
5,981711	0,027505	3,506212	1,869339	42,70376
0,019709	4,122307	0,206269	23,58736	29,39644
0,005145	0,178412	2,11252	7,874319	54,97876
0,459198	0,276948	2,57473	4,288726	42,62897
0,010526	0,003466	31,99932	3,764201	28,08478
0,1068	0,001086	4,916862	20,6418	26,32936
0,516284	0,014645	0,19882	14,03728	41,52669
0,113698	0,232008	1,915411	11,11151	34,62234
0,03004	0,123307	0,020586	0,307721	35,39721
0,042295	1,550253	1,300537	2,494645	44,74699
0	2,079174	0,252437	0,529991	19,22172
0,004277	0,069724	5,969464	0,445274	36,50196
0,13414	0,015209	15,18095	0,025011	15,59188
0,008833	0	6,949425	8,656009	27,06615
3,84925	0	0,682358	0,691322	47,85029
1,465406	0,150445	0,877621	22,98564	26,31871
0,006791	0	9,219166	4,32502	19,55991
0	0,254767	8,159179	3,094586	30,80716
1,320283	0,001799	3,301511	6,038009	28,4867
0,658488	0,001103	11,48245	6,543474	21,72539
0	0,00207	0,034216	11,12338	34,24569
0	0,159011	0,059783	0,334638	33,06505
0	0,098455	1,514623	3,44775	26,18773
0,047132	0,158151	0,017966	1,62013	20,36055
0,001463	0,050899	2,240205	1,870888	22,7603
0	3,480913	2,890144	6,959712	35,92124
0,447031	3,738666	4,737189	10,13596	35,65469
0,100413	2,027463	4,856738	3,350849	44,59635
0	0,045416	2,089341	7,760207	26,43055
0	1,050137	6,763244	1,867823	16,99617
0,120204	0,103732	2,844792	25,05018	26,4186
0,580305	1,484244	0,122909	3,96781	19,46133
1,052147	0,006788	0,317533	0,191666	33,51731
0,136884	2,099197	5,018824	8,896834	15,52652
3,615171	3,546129	4,514938	4,875818	47,58687
0,129036	0,009717	7,663311	0,337524	56,45979
4,94351	1,078076	0,908841	3,391549	46,44367
0,790289	0	0,201321	1,874529	68,43764
0	0,028161	12,99197	0,239571	38,87985
0,001043	0,005374	6,002881		62,04173
0				
0,106232				
0,009958				
0,039281				
0,04461				
0,496537				
0,717406				

Tail Intensities N3

Con	LY	HU	LY+HU	t-BOOH
0,1181	0,060479	0	2,702388	31,64886
0	0,128912	4,894863	0,38161	17,34053
0,191172	0,038716	4,998686	0,056068	45,05015
1,921582	2,808409	4,062397	0,363354	24,14726
0	0,405061	0,406002	31,66671	41,31493
0,00778	0,099643	0	0,781424	43,37153
0,004316	0,061739	16,91711	1,344776	38,4164
0,001499	0	3,731128	2,33075	32,37333
1,557201	0,011363	0,496153	2,756454	26,42538
0	0,011918	2,765136	20,64813	36,13902
0,179902	1,786117	12,4026	0,458722	40,59843
0,548768	0,104062	0,005505	0,122154	33,96783
0,059881	0,006313	0,02793	3,659955	28,16019
0,000912	0,004628	0,014524	0,230831	37,48395
0,08509	0,192597	2,146115	1,733476	30,07023
0,401865	0,002615	0,964962	2,052305	23,83434
0,010094	0,228729	0,028486	0,006828	44,06525
0,054374	0,064302	0,046598	1,018375	30,58472
0,02892	0,804106	4,680607	0,074773	18,57641
0,001539	0,050831	4,498522	3,978154	55,00641
0,106333	0,604907	3,899161	0,028198	39,8519
2,68854	0	5,061463	7,382128	23,64072
0,033426	1,45805	2,709388	14,03575	29,55814
0	1,547101	1,843029	13,2247	36,99881
0,001438	0,011816	2,190539	0,310574	40,38026
0,000736	0,053284	3,933158	5,481355	30,32238
2,344682	0,007713	0,542483	3,88244	36,99463
0,019053	0,789395	2,583361	13,03104	36,72874
0,111753	0,307264	1,201298	11,58167	46,30097
2,930859	0,076648	8,430606	0,038454	45,04566
0	1,545214	2,031566	0,911081	39,07838
0,131912	0,569155	3,412787	0,326509	37,64995
0,338709	0,070943	3,050919	0,384859	40,60642
3,102084	0,312556	1,435623	0,10184	46,47538
0,061503	2,390717	4,490301	1,50463	29,06651
1,064383	0,908709	0,679091	4,033714	37,08065
1,739837	0,032609	0,567138	0,04641	40,36659
0,026894	0	0,89027	0,760111	25,5757
2,002386	1,078525	5,035518	14,4027	34,38102
3,836015	0,378715	0,640123	1,187312	18,35437
1,786397	0,001475	0,422187	22,62207	33,05182
0,001012	0,095547	0,001327	5,583479	24,16502
0,002044	0,023881	0,083514	15,43479	36,13715
0	0,018409	1,308701	20,76724	54,55738
1,744184	0,105665	0,244578	21,05621	39,01974
1,137027	0,030744	0,00028	1,96799	59,46437
0,011762	0,053729	10,54311	0,33135	32,19831
0,991772	0	4,27125	3,072043	35,70308
4,733827	0,00133	0,337246	10,66096	37,12002
0,013009	0,861773	0,324766	0,286811	23,37235
0,000839	0	8,358777	4,523536	40,95492
0,000925	0,026926	0,687724		39,56779
0,287972	0,881062	3,754616		31,38338
0,6134	3,32661	2,193539		
0,091691	0,094503	2,571267		
1,787943		0,05471		
0,00034				

Original blots corresponding to Fig. 3A

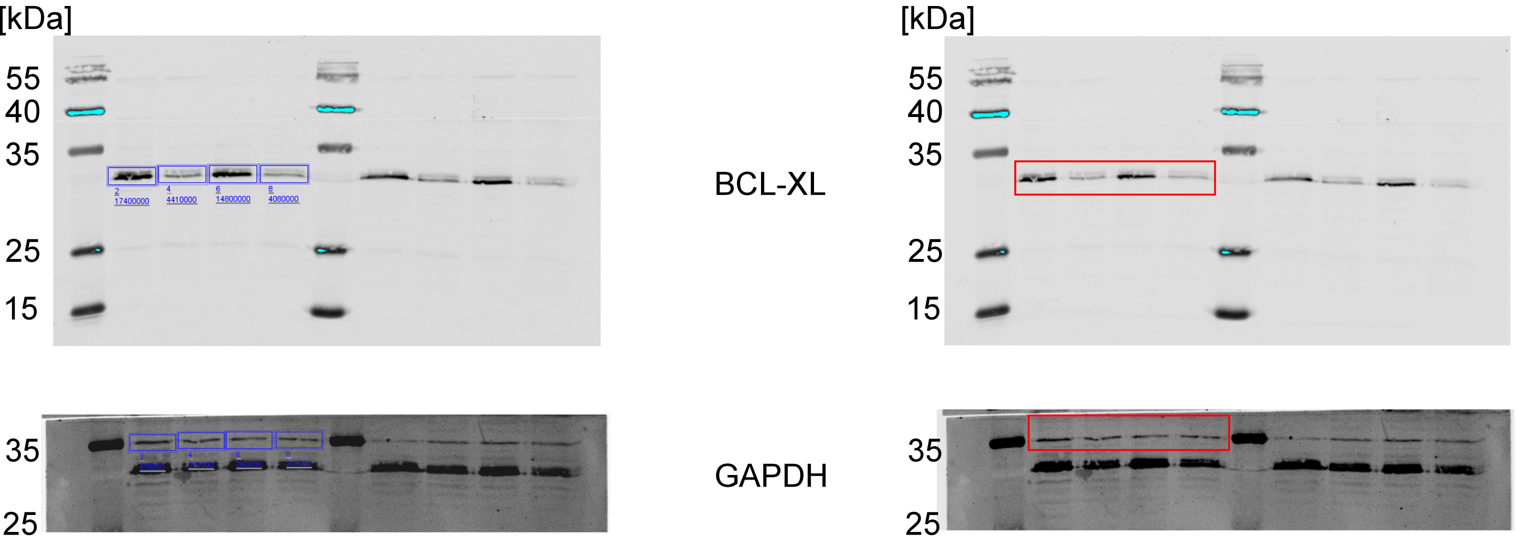

Original blots corresponding to Fig. 3D

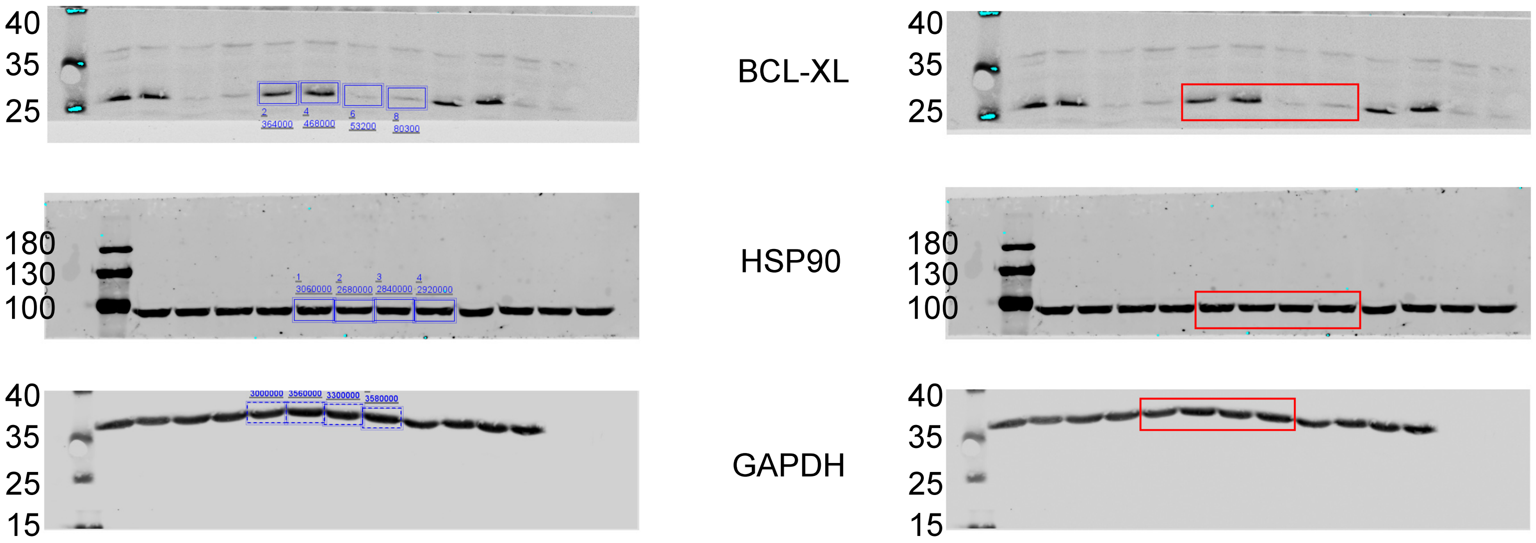

Figure 3A

	BCL-XL		GAPDH	
	Signal	Ratio	Signal	Ratio
con	17400000	1,00	476000	1,00
1 µM LY3009120	4410000	0,25	294000	0,62
1 mM HU	14800000	0,85	280000	0,59
LY3009021 + HU	4080000	0,23	265000	0,56

Figure 3D

	BCL-XL		HSP90		GAPDH	
	Signal	Ratio	Signal	Ratio	Signal	Ratio
siCon - Con	364000	1,00	3060000	1,00	3000000	1,00
siCon - 1 mM HU	468000	1,29	2680000	0,88	3560000	1,19
siBCL-XL - Con	53200	0,15	2840000	0,93	3300000	1,10
siBCL-XL - 1 mM HU	80300	0,22	2920000	0,95	3580000	1,19

Original blots corresponding to Fig. 4A

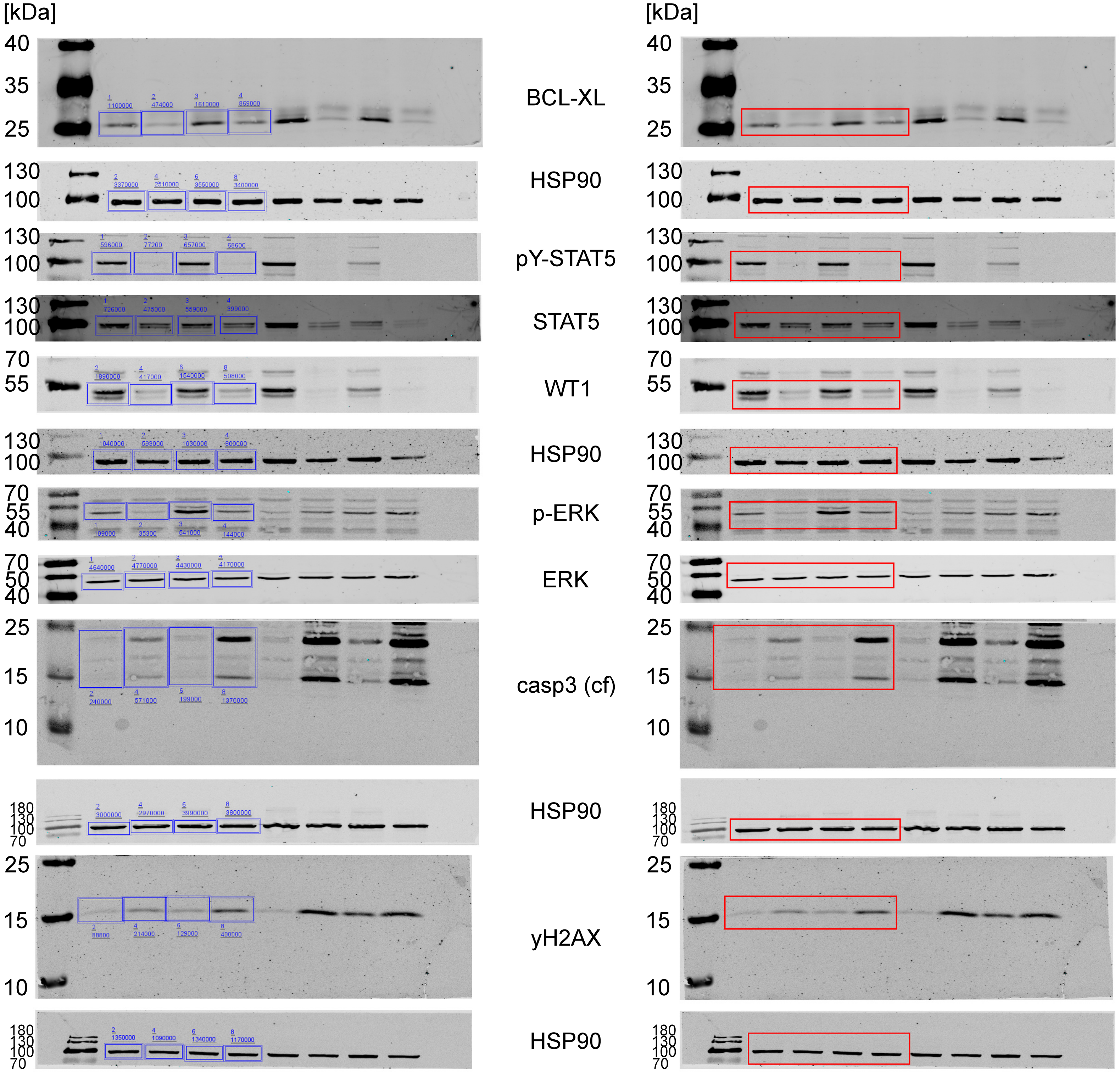

Figure 4A

	BCL-XL		HSP90		pY-STAT5		STAT5		WT1		HSP90	
	Signal	Ratio	Signal	Ratio	Signal	Ratio	Signal	Ratio	Signal	Ratio	Signal	Ratio
con	1100000	1,00	3370000	1,00	596000	1,00	726000	1,00	1890000	1,00	1040000	1,00
1 µM imatinib	474000	0,43	2510000	0,74	77200	0,13	475000	0,65	417000	0,22	593000	0,57
1 mM HU	1610000	1,46	3550000	1,05	657000	1,10	559000	0,77	1540000	0,81	1030000	0,99
imatinib + HU	869000	0,79	3400000	1,01	68600	0,12	399000	0,55	508000	0,27	800000	0,77

	p-ERK		ERK		casp3 (cf)		HSP90		yH2AX		HSP90	
	Signal	Ratio	Signal	Ratio	Signal	Ratio	Signal	Ratio	Signal	Ratio	Signal	Ratio
con	109000	1,00	4640000	1,00	240000	1,00	3000000	1,00	88800	1,00	1350000	1,00
1 µM imatinib	35300	0,32	4770000	1,03	571000	2,38	2970000	0,99	214000	2,41	1090000	0,81
1 mM HU	541000	4,96	4430000	0,95	199000	0,83	3990000	1,33	129000	1,45	1340000	0,99
imatinib + HU	144000	1,32	4170000	0,90	1370000	5,71	3800000	1,27	400000	4,50	1170000	0,87

Original blots corresponding to Fig. 5A

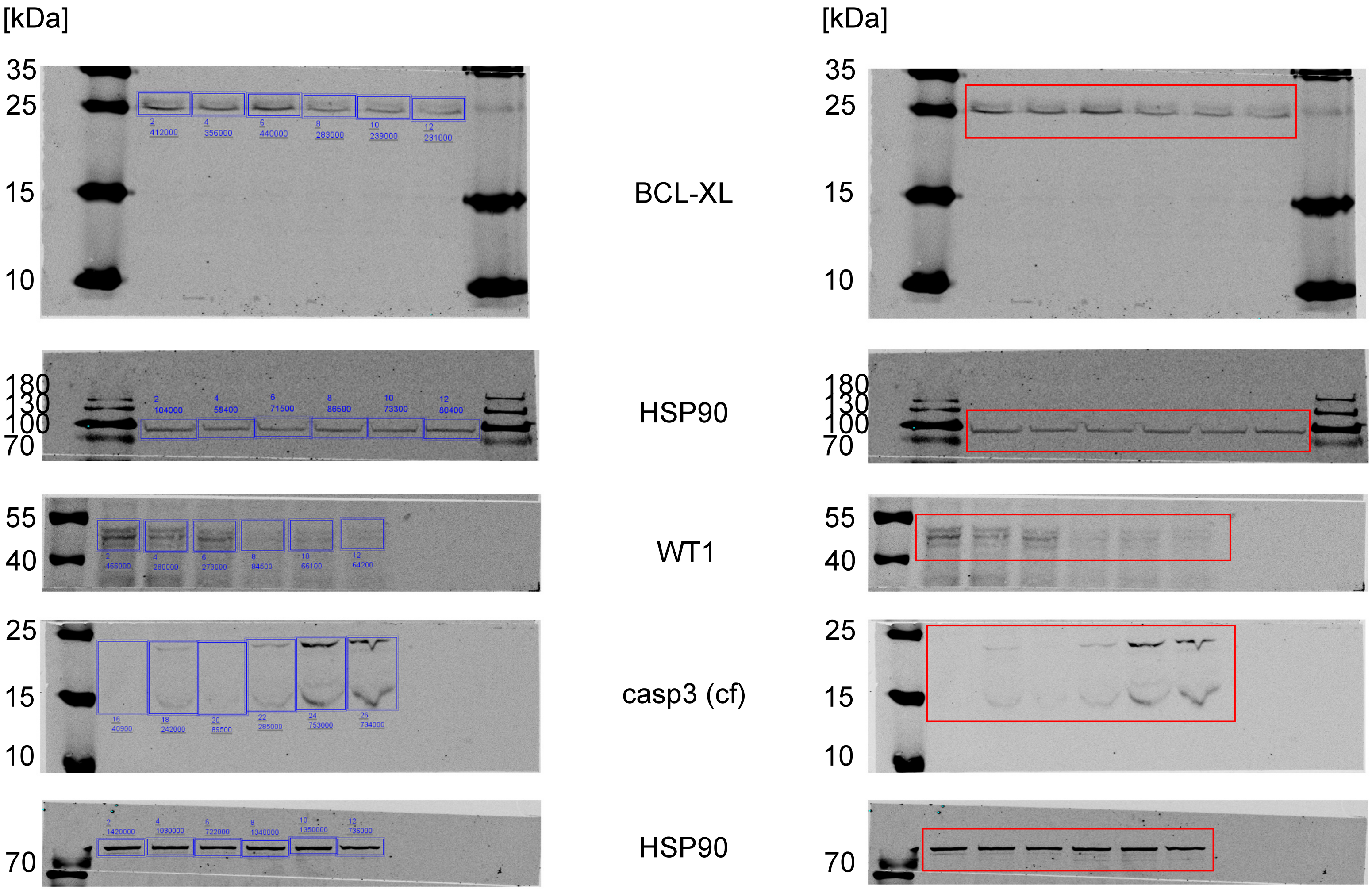

Figure 5A

	BCL-XL		HSP90		WT1		casp3 (cf)		HSP90	
	Signal	Ratio	Signal	Ratio	Signal	Ratio	Signal	Ratio	Signal	Ratio
con	412000	1,00	104000	1,00	466000	1,00	40900	1,00	1420000	1,00
0.5 mM HU	356000	0,86	59400	0,57	280000	0,60	242000	5,92	1030000	0,73
0.5 µM LY3009120	440000	1,07	71500	0,69	273000	0,59	89500	2,19	722000	0,51
1 µM LY3009120	283000	0,69	86500	0,83	84500	0,18	285000	6,97	1340000	0,94
HU + 0.5 µM LY300912	239000	0,58	73300	0,70	66100	0,14	753000	18,41	1380000	0,97
HU + 1 µM LY3009120	231000	0,56	80400	1,35	64200	0,14	734000	17,95	735000	0,52

Original blots corresponding to Fig. 6A

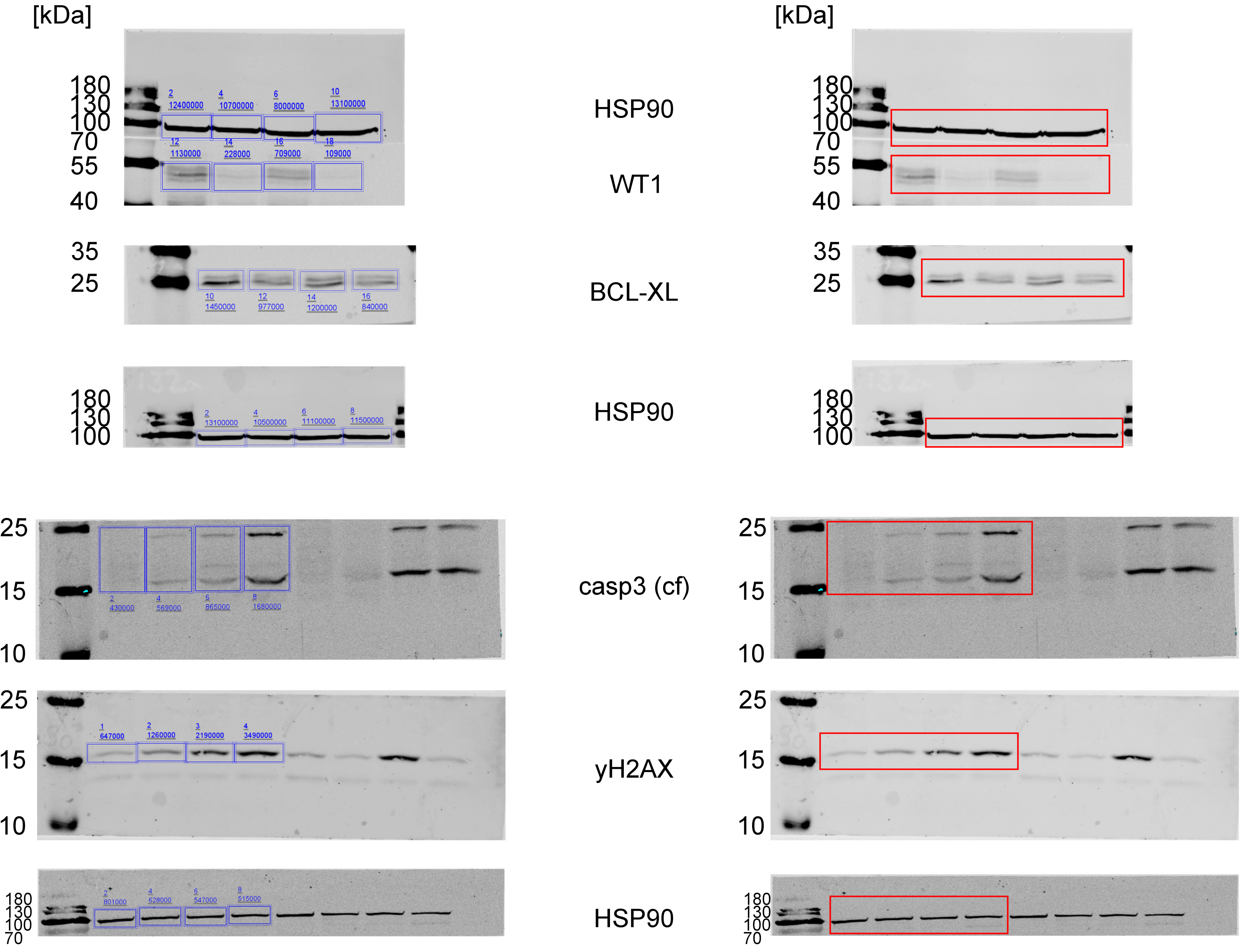

Figure 6A

	WT1		HSP90		BCL-XL		HSP90	
	Signal	Ratio	Signal	Ratio	Signal	Ratio	Signal	Ratio
con	1130000	1,00	12400000	1,00	1450000	1,00	13100000	1,00
2 nM quzartinib	228000	0,20	10700000	0,86	977000	0,67	10500000	0,80
0.5 mM HU	709000	0,63	8000000	0,65	1200000	0,83	11100000	0,85
quizartinib + HU	109000	0,10	13100000	1,06	840000	0,58	11500000	0,88

	casp3 (cf)		yH2AX		HSP90	
	Signal	Ratio	Signal	Ratio	Signal	Ratio
con	430000	1,00	647000	1,00	801000	1,00
2 nM quzartinib	569000	1,32	1260000	1,95	628000	0,78
0.5 mM HU	865000	2,01	2190000	3,38	547000	0,68
quizartinib + HU	1680000	3,91	3490000	5,39	515000	0,64

Original blots corresponding to Fig. 7A

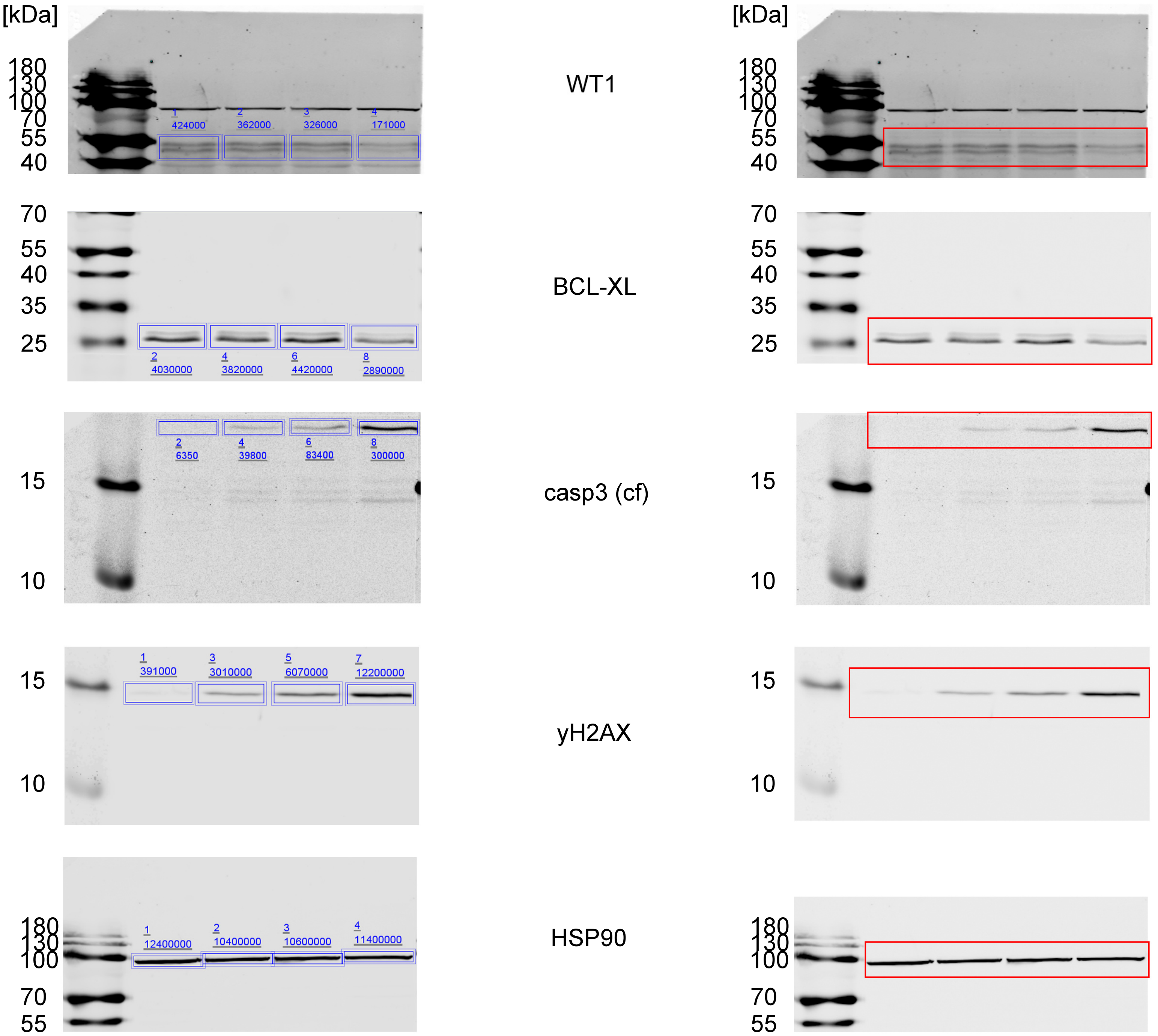

Figure 7A

MV4-11	WT1		BCL-XL		casp3 (cf)		yH2AX		HSP90	
	Signal	Ratio	Signal	Ratio	Signal	Ratio	Signal	Ratio	Signal	Ratio
con	424000	1,00	4030000	1,00	6350	1,00	391000	1,00	12400000	1,00
0.5 mM HU	362000	0,85	3820000	0,95	39800	6,27	3010000	7,70	10400000	0,84
0.5 uM Navitoclax	326000	0,77	4420000	1,10	83400	13,13	6070000	15,52	10600000	0,85
0.5 mM HU + 0.5 uM Navitoclax	171000	0,40	2890000	0,72	300000	47,24	12200000	31,20	11400000	0,92
